# Plasmonic vortices for tunable manipulation of target particles, using arrays of elliptical holes in a gold layer

**DOI:** 10.1038/s41598-022-27109-7

**Published:** 2023-01-02

**Authors:** Amir Mohammad Ghanei, Abolfazl Aghili, Sara Darbari, Nahid Talebi

**Affiliations:** 1 Nano-Sensors and Detectors Lab., and Nano Plasmo-Photonic Research Group, Faculty of Electrical and Computer Engineering, Tarbiat Modares University, Tehran, Iran; 2 Institut für Experimentelle und Angewandte Physik, Kiel University, 24118 Kiel, Germany; 3 Kiel, Nano, Surface, and Interface Science-KiNSIS, Kiel University, 24098 Kiel, Germany

**Keywords:** Engineering, Optics and photonics, Physics

## Abstract

Here, we numerically prove that light with linear polarization can be coupled to surface plasmon polaritons at an elliptical hole perforated in a gold layer to generate plasmonic vortex (PV). Benefiting from the smooth variation of the minor to major ellipse axes, a gradual variation in the phase profile of the generated PV is achieved. Regarding this, three types of independent arrays of elliptical holes are presented, which can produce uniform and high quality PVs with different topological charges at the center of the arrays. The first array can produce PV with topological charges of + 1 and − 1, depending on the polarization orientation of the incident light. In the second one, the topological charge of the PV can be switched between 0 and + 2, by switching the polarization direction of the incident light. In the third array, a robust PV with topological charge of + 1 is generated independent of possible tolerances in the polarization orientation. In order to use the generated PVs for plasmonic tweezing application, there are side fringes around the central vortex of the arrays that should be eliminated. To produce a single vortex, we propose metal-insulator-metal (MIM) structures, screening excessive fringes and allowing the central PVs to leak out. It is also demonstrated by simulation that target particles, such as gold and polystyrene spheres of subwavelength dimensions, can be efficiently manipulated by our MIM designs, suitable for different applications including local mixing, and applying switchable torque or force to target particles to explore their complete elastic characteristics.

## Introduction

Almost three decades ago, Allen et al.^1^ introduced the optical wave carrying orbital angular momentum (OAM), which is called an optical vortex (OV). OVs are waves representing a phase singularity and a rotational field around a dark spot, and these waves show a helical wavefront and a spiral Poynting vector around the axis of propagation^2^. The OV phase is expressed by ${e}^{il\varphi }$ mathematically, wherein $\varphi$ is the azimuthal angle of the vortex field and *l* is the topological charge determined by the number of phase discontinuities within a full azimuthally rotation, and can take integer or fractional values. OV has attracted great attention in application fields such as optical communications^3–5^, data processing^6,7^, optical trapping^8^ and rotating microparticles^9,10^. Various methods have been proposed to produce OV, including helical phase plates^11–13^, spatial light modulators^14,15^, computer holography^16^, optical waveguides^17^, and metasurfaces^18–21^. However, conventional OV generators suffer from the bulky configuration and limited performance.

On the other hand, near-field OAM, supported for example by a plasmonic vortex (PV), has attracted remarkable attentions and interests. These PVs are typically generated by the excitation of surface plasmon polaritons (SPPs) at the interface of a metal and a dielectric. PVs have inherently strong optical angular momentum in their evanescent field region^22^; thus, it is efficiently applicable to plasmonic tweezers^23–26^, data storage^27^ and solitons^28^. PVs can be generated in various plasmonic geometries including plasmonic Archimedes spirals^29–33^, plasmonic rings^34,35^ and plasmonic slits^36,37^. The required polarization of illumination in PVs depends on the plasmonic structure, although circularly and radially polarized illuminations are the most common^33,35,38^. Among these, in radially polarized sources the PV behavior relies on complicated source embedments. Moreover, generation of PVs by circularly polarized source is based on converting an incident far-field spin angular momentum (SAM) to a near-field OAM through excitation of plasmonic structures. Recently, generation of PVs has been demonstrated by linearly polarized illumination in some specific geometries such as, asymmetric cross nanocavities in a gold layer^39^, rectangular holes^40^, and single elliptical nanoholes^41^. Benefiting from inherently strong spatial confinement of plasmonic fields in PVs, we can overcome the diffraction limit in conventional optical tweezers^42^, which allows for manipulating target particles with subwavelength sizes^43–50^. Moreover, manipulating metallic nanoparticles by OVs has been proved to be challenging due to strong absorption and scattering of metallic particles in a focused optical beam. In contrast, plasmonic tweezers and consequently PVs have been shown to be capable of trapping and rotating of metallic particles in a stable manner^51^.

Here, we propose elliptical holes in a gold layer as efficient sources for generating PVs by linearly polarized illumination. Three different configurations of arrays of elliptical holes are also suggested to generate higher quality PVs with different topological charges. Two of the structures are optimized in a way to be able to switch the topological charge order by the orientation of the polarization of the incident light. However, the proposed single stage arrays suffer from intense side plasmonic fields other than the main central PV, hindering its application in manipulating the target particles by PVs. Therefore, in the next step, MIM configurations are recommended in each design, in which the proposed elliptical hole arrays serve as the bottom stage PV source, where a circular hole in the top gold layer allows this PV to be extracted, and interact with the target particles exclusively. The designed PVs benefit from central, uniform and high-quality vortices, which can be controlled by the incident polarization angle and allow uniform and smooth rotating manipulation functionalities.

## Simulation models and methods

To measure the plasmonic force exerted on the particle, first, the electric and magnetic fields should be calculated around the particle in the vicinity of the metallic layer by 3D finite-difference time-domain (FDTD) simulation method^52^. Then, the averaged plasmonic gradient and scattering forces exerted on the target particle are found by Maxwell’s stress tensor (MST)^53,54^ given by

1 $$\langle F\rangle =\frac{1}{2}Re{\oint }_{\Omega }T\left(r,t\right).\widehat{n}dS,$$

wherein the Maxwell’s stress tensor (T) recast as

2 $$T\left(r\right)=\varepsilon E\left(r\right)\otimes {E}^{*}\left(r\right)+\mu H\left(r\right)\otimes {H}^{*}\left(r\right)-\frac{1}{2}(\varepsilon {\left|E\left(r\right)\right|}^{2}+ \mu {\left|H(r)\right|}^{2})$$

Here,* ε* and *μ* denote the medium permittivity and permeability, **E** and **H** represent the time-harmonic electric and magnetic field vectors, **r** signifies the position vector, $\otimes$ is the tensor product, Ω is the cubic volume that surrounds the particle, moving along with the particle movement, and **n** is the unitary vector perpendicular to the surface *S,* which encloses Ω. Boundary conditions along all axes are assumed to be perfectly matched layers (PMLs) in our simulations^55,56^. Furthermore, the trapping potential profiles are acquired by integrals ${U}_{x}=\int {F}_{x}dx$ and ${U}_{y}=\int {F}_{y}dy$, where ${F}_{x}$ and ${F}_{y}$ are *x*-component and *y*-component of the plasmonic force, respectively. Dispersion behavior of the gold dielectric constant is extracted from the literature, and is utilized in our simulations^57^.

## Plasmonic modes around a single elliptical hole under linearly polarized excitation

**Figure 1:**
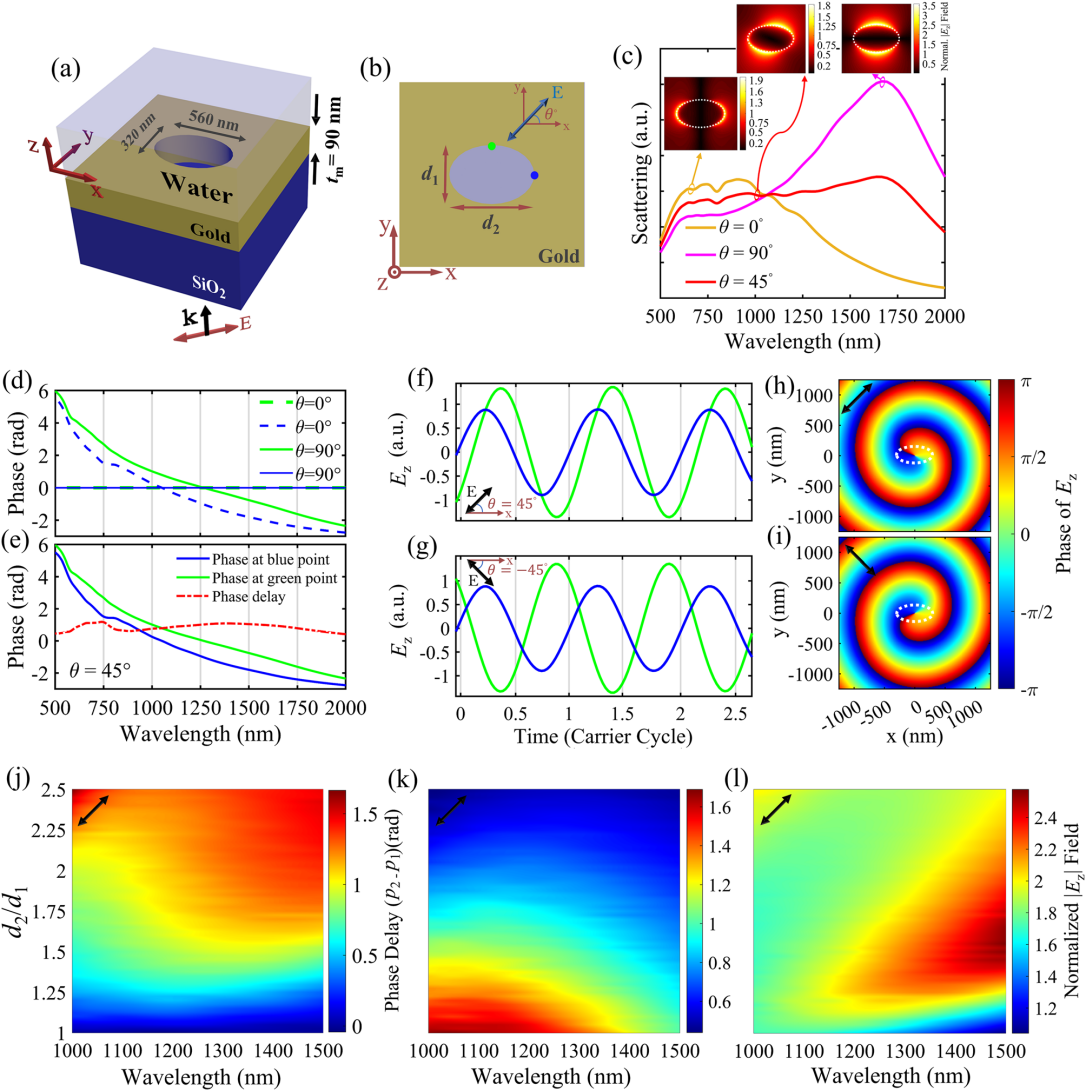
(**a**) Three-dimensional and (**b**) top-view (x–y plane) schematic diagrams of an elliptical hole embedded in a gold layer deposited on the glass substrate. *d*_1_ and *d*_2_ are the minor axis and the major axis of the elliptical hole, respectively. (**c**) The scattering spectrum of a single elliptical hole measured in different *θ* values of 0°, 90° and 45°. Dimensions of the hole are *d*_1_ = 320 nm and *d*_2_ = 560 nm. The insets show corresponding normalized spatial distribution of the *E*_z_ components. (**d**,**e**) calculated phase of *E*_z_ field under *θ* = 0°, 90° and 45° at two observation points marked by blue and green dots in (**b**). (**f**,**g**) The relative amplitude and phase relationships between green and blue points for *θ* = 45° and − 45°, respectively, for *λ*_0_ = 1047 nm. (**h**,**i**) the phase distribution of *E*_z_ field in 2π range around a single elliptical hole for *θ* = 45° and − 45°, respectively. White dashed lines illustrate the elliptical hole boundaries. (**j**) Calculated phase delay of *E*_z_ between blue and green points as a function of *d*_2_/*d*_1_ and wavelength, for *θ* = 45°. Here, *P*_2_ and *P*_1_ represent phase values at green and blue points. (**k**,**l**) Calculated instantaneous normalized *E*_z_ field, for *θ* = 45° as a function of *d*_2_/*d*_1_ and wavelength, at blue and green points respectively. Black arrows in parts (**f**–**l**) indicate polarization orientations. All simulation results are probed at 10 nm above the gold layer, in water medium.

To overcome the complexity of realizing circular and radial incident polarizations, designing a plasmonic structure, capable of generating PV by a linearly polarized incident beam is proposed. For this purpose, we consider exciting SPs of subwavelength apertures with asymmetrically elongated shape, such as rectangular or elliptical holes. Here, elliptical holes are chosen due to their geometrical smooth transition between major and minor axes, leading to a smooth plasmonic field variation in response to rotating orientation of the polarization of an incident linearly polarized light. This behavior can lead to a consistent distribution of the gradient force around the elliptical hole, as compared with other asymmetric counterparts such as rectangular and cross-shaped holes. Figure 1a,b show the schematic 3D and top views of a single elliptical hole, which is embedded in a gold layer with thickness of *t*_m_ = 90 nm on a glass substrate, and the top ambient medium is water. Materials and dimensions of the optimal elliptical hole structure are presented in Fig. 1a, wherein a TM polarized plane wave with wave vector (*k*) along the *z*-direction is assumed as the incident wave. The minor (*d*_1_) and major (*d*_2_) axes of the investigated single elliptical hole (Fig. 1b) are assumed 320 nm and 560 nm, respectively. Here, *θ* is defined as the polarization angle with respect to the *x*-axis, as illustrated in Fig. 1b. To study the plasmonic behavior of the single elliptical hole, the calculated scattering spectra corresponding to polarization angles of 0°, 45°, and 90° are plotted in Fig. 1c. The insets display the normalized *E*_z_ component of electric field distributions, corresponding to plasmonic peaks of each spectrum. For *θ* = 90°, a wide plasmonic peak at 1700 nm is observable, which corresponds to excitation of the electric dipole along the minor axis of the ellipse. In contrast, when the structure is excited with the polarization direction corresponding to *θ* = 0°, a damped broad peak at shorter wavelengths is observed. Intuitively, one could expect the opposite behavior, since electric dipolar excitations along the major axis scatter the light at longer wavelengths compared to those electric dipoles excited along the minor axis. Nevertheless, it is well known that for void plasmonic structures, the dominating scattering mechanism is due to the excitation of magnetic multipoles (here dipoles), in contrast with nanoparticles where induced electric multipoles make the major contribution. Such a behavior could be deduced from the generalization of the Babinet’s principle for plasmonics^58,59^ (see Fig. S1). For *θ* = 45° we can partially excite both the discussed perpendicular dipole oscillators, as shown in the inset of Fig. 1c. The corresponding scattering spectrum (red spectrum) also seems as the superposition of the purple and yellow spectra, which approximately covers the whole wavelength range (700 nm to 1700 nm). It is worth noting that in near-field diffraction, the longitudinal component of the field (*E*_z_) is the prominent part of it, and in-plane magnetic fields dominate due to the effective magnetic resonances; So, in our study, in order to understand the behavior of the PV, we only consider the phase distribution of *E*_z_. When *θ* = 0°, as illustrated in Fig. 1d, the *z*-component of SPs at blue point that is shown in Fig. 1b, has a phase in a wide range of spectrum, while SPs at the green point are not excited, so it has no phase. Then, by changing *θ* to 90°, an electric dipole is generated along *y*-axis. If we adjust *θ* = 45°, in this case, SPs along both axes are excited simultaneously. As shown in Fig. 1e, the *z*-component of SPs at blue and green points have a phase, which in blue point precedes the green point in this range of wavelengths. The generated phase delay is also shown by the red curve in this figure.

Now, the phase of the *E*_z_ component of the excited oscillating dipoles is studied for *θ* =  ± 45° and *λ*_0_ = 1047 nm in the investigated single elliptical hole (*d*_1_ = 320 nm, *d*_2_ = 560 nm). For more clarification, the time-domain distribution of the *z*-component of SPPs are presented at two observation points along the ellipse axes (green and blue dots in Fig. 1b) for *θ* = 45° and − 45° in Fig. 1f,g, respectively. Here, the time scale has been normalized to one period cycle of the oscillating SPPs. The time resolved field investigation reveals a fixed phase delay between the fields at blue and green points, which are demonstrated by blue and green signals respectively. Tracing the phase delays shows that for *θ* = 45° (− 45°), the phase of *E*_z_ at green (blue) point always precedes, so that the PV will be counter-clockwise (clockwise). The observed phase delay and amplitude between the field signals depends on the ellipse axes dimensions. This phase delay originates from the excited dipoles with different effective lengths, oscillating upon equivalent perpendicular slits with different widths (along *x* and *y* directions). From the operational view, elliptical holes can be assumed equal to a circular hole and a quarter-wave plate in series. They both convert the polarization state of the impinging light from the linear to elliptical by introducing a phase delay between perpendicular field components. However, it is notable that our design avoids the complicated and bulky structure of quarter-wave plates, and benefits from a compact structure, without any significant fabrication challenge. Moreover, Fig. 1e reveals that the presented elliptical hole benefits from a nearly uniform phase delay in a wideband wavelength range (500–2000 nm), the observation which is in accordance with the presented wideband plasmonic behavior for *θ* = 45° in Fig. 1c. As expected, the closer-values of ellipse axes dimensions, or equally a more symmetric elliptical hole, leads to less phase delay and closer amplitudes of *z*-component fields. The observed phase delay in *E*_z_ components leads to a rotational plasmonic field on the Au/water interface, which is called a PV. This PV can exert a rotational scattering force to the target particles suspended in water, and rotate the particles. It is notable that closer values of the axes of the ellipse lead to less phase delay with weaker PV behavior, but closer amplitude values of the *E*_z_ components with smoother rotational behavior of the target particle. Figure 1h,i indicate the *E*_z_ phase distributions at *λ*_0_ = 1047 nm, confirming the counter-clockwise PV (*l* =  + 1) for *θ* = 45°, and clockwise PV (*l* = − 1) for − 45°.

Furthermore, the ellipse axes dimensions are optimized for generating an efficient PV by monitoring the *E*_z_ phase delay between the green and blue (*p*_2_ – *p*_1_) points (Fig. 1j), and normalized amplitude of z-component of SPPs at blue (Fig. 1k) and green (Fig. 1l) points. Therefore, we elaborate on the effect of ellipse axes ratio and incident wavelength on these parameters, for *θ* = 45°. We kept *d*_1_ = 320 nm fixed, and swept *d*_2_ from *d*_1_ to 2.5*d*_1_. It is apparent in part (j) that at a specific wavelength, a higher *d*_2_/*d*_1_ ratio leads to a higher phase delay. By utilizing these plots, the best dimensions for the elliptical hole can be chosen at a desired incident laser wavelength. For example, by assuming incident wavelength of 1047 nm and *θ* = 45°, the chosen elliptical dimensions (*d*_1_ = 320 nm, *d*_*2*_ = 560 nm) lead to a phase delay of almost 0.85 rad, and *E*_z_ ratio of 1.35 for the green point with respect to the blue point.

Based on the aforementioned plasmonic response of the elliptical hole, in the following section, three different array designs with different topological charges are presented for plasmonic tweezing, capable of trapping and/or rotating the desired target particles.

## Functional plasmonic arrays with different topological charges

Although the topological charges of ± 1 have been already achieved by exciting a single elliptical hole with polarization angles *θ* =  ± 45°, we can still enhance the PV characteristics for application in plasmonic tweezers, by designing appropriate hole arrays. Elliptical hole arrays can lead to more uniformity, consistency and circular symmetry in the resulting plasmonic field and uniform rotations of the target particles. On the other hand, we are restricted to topological charges of ± 1 in a single elliptical hole, resulting in a low degree of controlling over particle manipulation. Therefore, we prove achieving various topological charges in the arrays of elliptical holes, including 0, ± 1, 2, suitable for controllable trapping, and clockwise/anticlockwise rotations. Within arrays of elliptical holes, each hole behaves as an independent plasmonic vortex source. Superposition of these plasmonic vortex sources, reveals an equivalent plasmonic vortex with higher quality, uniformity and controllability. Now, our array designs with different geometries are presented to generate PVs with different topological charges, utilizing a linearly polarized incident wave for the first time, then prove their different plasmonic manipulation functionalities.

### Designing an elliptical hole array (I) with switchable rotating of the trapped particles

Here, we design an array of elliptical holes capable of bidirectional rotation of a plasmonic vortex, depending on the polarization orientation of the incident light. According to our previous discussions, the array can de designed to generate vortices with different topological charges. Among different geometric designs for the array, the Archimedes spiral can be utilized^33,39^, which is well known for generation of plasmonic vortices with different topological charges, depending on the design parameters. The main design parameter of the Archimedes spiral is the distance between the spiral and the origin at each point of the spiral curve (*r*), which is expressed in the cylindrical coordinate system as

3 $$r\left(\varnothing \right)={r}_{0}+\frac{m\varnothing {\lambda }_{SPP}}{2\pi },$$

wherein, *r*_0_ is the initial radius, ∅ is the azimuthal angle associated with the geometry, and *m* is the geometrical charge that can take any integer value. Positive (negative) *m* values lead to right-handed (left-handed) Archimedes spirals. Moreover, *λ*_SPP_ is defined as the SPP wavelength and given by

4 $${\lambda }_{SPP}={\lambda }_{0}{(\frac{{\varepsilon }_{d}+{\varepsilon }_{m}}{{\varepsilon }_{d}{\varepsilon }_{m}})}^{1/2},$$

where, *λ*_0_ is the incident wavelength, ${\varepsilon }_{d}$ and ${\varepsilon }_{m}$ are the permittivities of dielectric and metal, respectively. As discussed in Fig. 1h,i, each single elliptical hole can generate a PV with topological charges of *l* = $\pm 1$ depending on the polarization orientation of the incident light, which is added to the geometrical charge of Archimedes spiral (*m*) to generate the Archimedes spiral topological charge (*q*), as described in Eq. (5). In other words, each elliptical hole in the Archimedes spiral array with geometrical charge of *m* induces a geometrical phase delay to its SPPs, leading to a super positioned Archimedes PV at the center of spiral^37^:

5 $$q=m+l,$$

**Figure 2:**
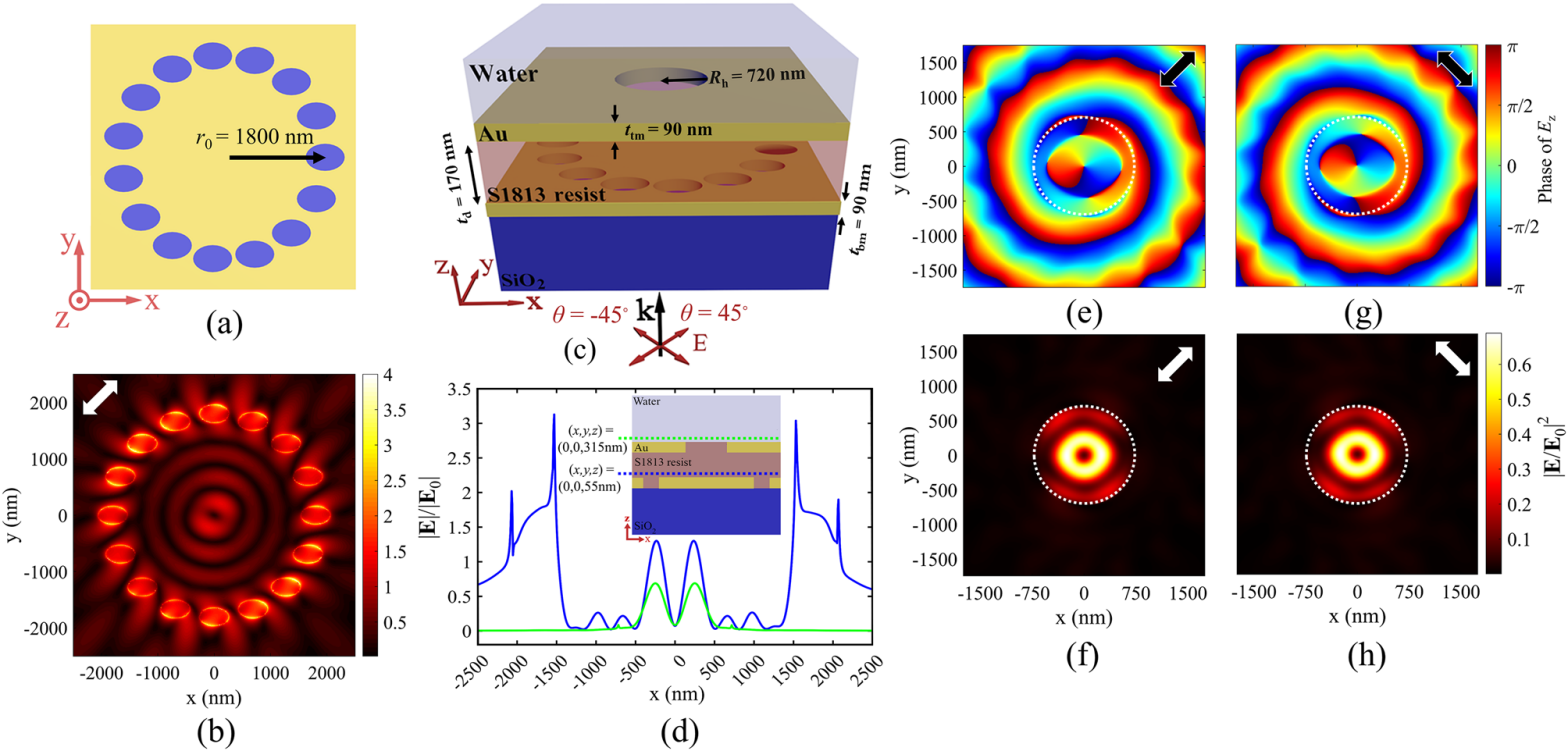
(**a**) Two-dimensional schematic diagram of a circular array of elliptical holes embedded in a gold layer, and (**b**) the relating normalized electric field distribution for *θ* = 45°. (**c**) Three-dimensional scheme of the proposed MIM structure (design I). (**d**) Normalized electric field along *x*-axis at *y* = 0, for *z* = 55 nm (green curve) and *z* = 315 nm (blue curve). (**e**,**f**) Phase distribution of *E*_z_ and the normalized field intensity distribution for *θ* = 45°. (**g**,**h**) Phase distribution of *E*_z_ and the normalized field intensity distribution for *θ* = − 45°. White dashed circles illustrate the boundary of the central circular hole etched in the top metal layer. White and black arrows indicate orientations of incident linear polarization.

First, the simplest form of an Archimedes spiral with *m* = 0 is investigated. In this case, a circular array of elliptical holes is assumed, as shown in Fig. 2a, in which the major axes of all elliptical holes are aligned along the *x*-axis, and all the holes are arranged to form a circular array with radius of *r*_0_ = 1800 nm. We assumed 16 holes in our array, and also considered *r*_0_ = 3*λ*_SPP_, large enough to allow simpler near-field measurements^39^ in possible future experimental implementations. The array structure is illuminated by linearly polarized light with *θ* = 45° and *λ*_0_ = 1125 nm to achieve the best quality PV. Figure 2b illustrates the field distribution in the *x*–*y* plane, 10 nm above the gold layer, proving successful excitement of PVs at each elliptical hole for *θ* = 45°. The excited plasmonic fields in the holes behave as plasmonic sources, which propagate and interfere to produce plasmonic circular standing waves in the central zone of the array. Due to the significant plasmonic losses, the field intensity decreases, as plasmons propagate towards the array center. Since *m* = 0 in this array, the propagation length and the phase delay of the plasmonic fields, from each PV source (elliptical holes) to the array center, are equally constant. Thus, the phase distribution of the array PV at the array center is only dependent on the phase profile of the plasmonic field generated by each elliptical hole. Considering the lower intensity of the central PV of the array, in comparison with the excited individual PVs of each elliptical hole, we cannot benefit from the array-generated uniform PV in this bare configuration (Fig. 2a) for particle manipulation, because the plasmonic gradient forces move the target particles toward the initial vortices of elliptical holes. Thus, it is essential to block excessive fields from localized plasmonic fields at elliptical holes, and prevent exposure of the target particles to theses excited plasmonic sources. For this purpose, an MIM plasmonic waveguide is designed as the top part of a whole plasmonic tweezers configuration, as depicted in Fig. 2c. According to the schematic cross section in this figure, the designed circular array of elliptical holes serves as the bottom gold layer with the thickness of *t*_bm_ = 90 nm in the MIM structure, while the top gold layer with the thickness of *t*_tm_ = 90 nm consists of a single circular hole, aligned on top of the center of the bottom circular array. In this configuration, excited SPs propagate and interfere through the MIM waveguide, and the top central circular hole is designed so that only the resulting central array PV transmits through it, leading to a uniform PV above the top gold layer, to which the target particles are exposed. The designed structural parameters of this configuration are depicted in Fig. 2c, among which the circular hole radius (*R*_h_) is assumed 720 nm. Moreover, the S1813 resist is assumed as the insulator in our MIM configuration, which has negligible index dispersion in our wavelength range of study, so that a constant refractive index of 1.625 can be assumed for the resist in our simulations. It should be noted that the thickness of the insulator layer has been assumed *t*_d_ = 170 nm, so that only fundamental TM_0_ mode of the MIM waveguide can propagate, allowing a PV with considerable intensity on the circular hole of the top gold layer. On the other hand, a thinner insulator layer leads to a higher plasmonic loss, due to the lossy characteristic of the metallic layers. Thus, there is a trade-off between the mode confinement and the plasmonic propagation loss in our MIM waveguide^60^ and PV intensity on top of the circular hole, which is compromised for the insulator thickness of 170 nm^38,61^. The proposed structure is illuminated by a linear polarization with *θ* = 45° at *λ*_0_ = 1180 nm to achieve the best quality PV for the MIM configuration of design I. Figure 2d indicates the normalized electric field along *x*-axis at *y* = 0, for *z* = 55 nm inside the MIM waveguide (shown by the blue dashed line in the inset), and *z* = 315 nm outside the MIM waveguide (shown by the green dashed line in the inset). The inset shows the cross-section schematic (*x*–*z* plane) of the structure. The observed double peaks around *x* = 0 in both blue and green curves in Fig. 2d belong to the main central vortex, while the side field intensities are negligible in green curve, as compared with the blue curve, revealing the efficient functionality of the MIM structure to block the side plasmonic fields, and maintaining the central PV. It is evident that the PV intensity (green profile), which is extracted from the MIM configuration and exposed to the target particles, has been reduced in comparison with the initial bottom PV (blue profile). The side ripples between ± 500 nm < *x* <  ± 1200 nm in blue curve are related to the plasmonic standing waves inside the array, while the high peak intensities at *x* =  ± 1500 nm and *x* =  ± 2000 nm arise from the localized surface plasmons of the elliptical holes. In the investigated illumination conditions with *θ* =  + 45°, each elliptical hole generates a PV with topological charge of *l* =  + 1 (as discussed in Fig. 1), leading to a total PV of *q* =  + 1 for this array (*m* = 0), according to (5). Figure 2e,g shows phase distribution of *E*_z_ for the MIM configuration, when *θ* =  + 45° (− 45°), revealing a total PV with topological charge of *q* =  + 1 (− 1). Figure 2f,h illustrates the distribution of the normalized electric field intensity of the corresponding PVs. According to (5), *l* = − 1 (corresponding to *θ* = − 45°), evidently leads to *q* = − 1 and a clockwise rotating PV, as shown in Fig. 2g. The presented normalized electric field intensity distribution in Fig. 2f,h imply a highly uniform and annular total PV, as compared with the preliminary PVs generated from each individual elliptical hole (shown in the middle inset of Fig. 1c). It is notable that our simulated PV is in accordance with the analytical solution for the Archimedes array in cylindrical coordinate, leading to electric field components with the spiral phase profile of $q\varphi$^22^.

**Figure 3:**
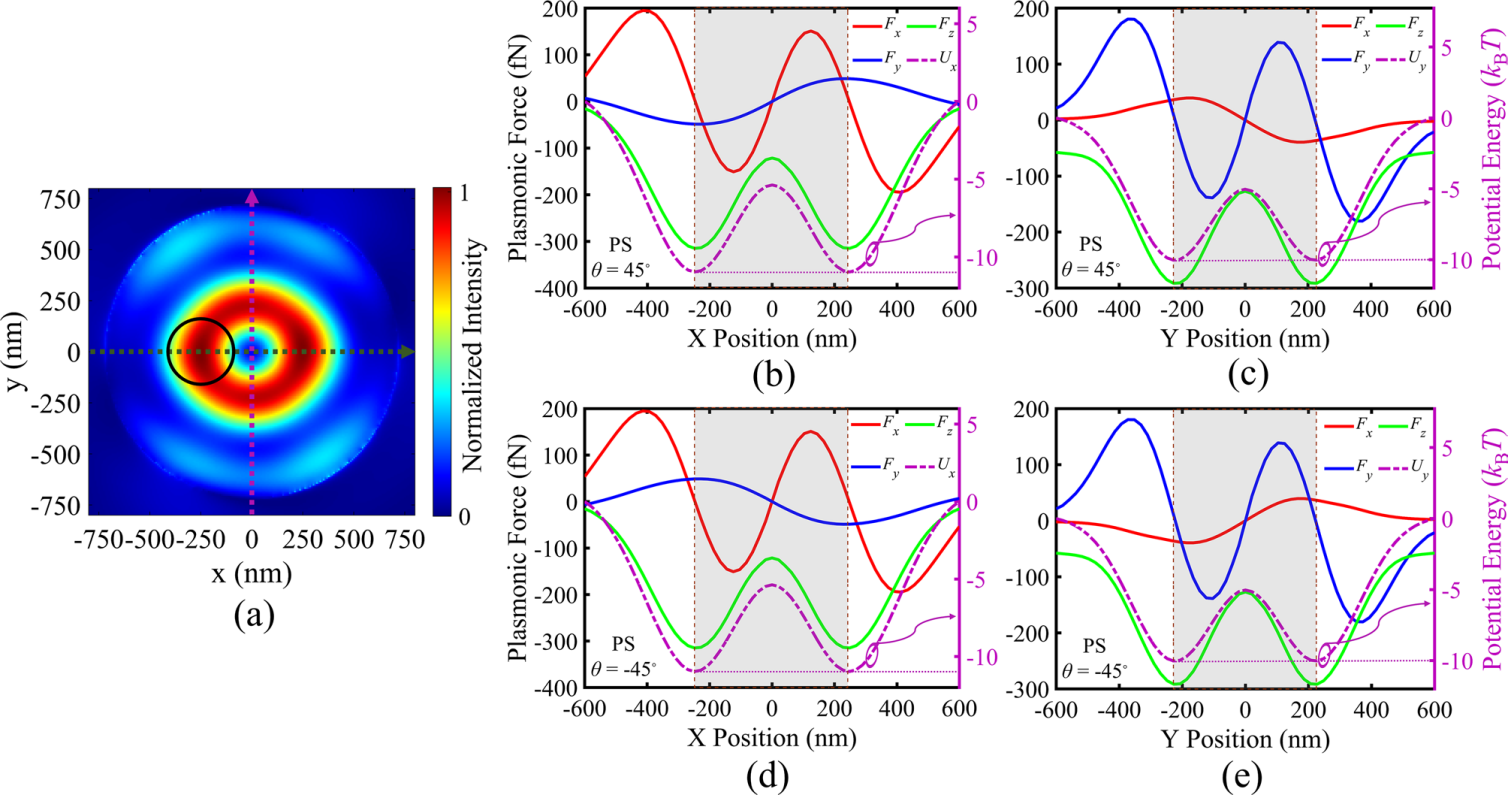
(**a**) Directions of PS particle movement tracks with respect to the normalized field intensity distribution of the generated PV in MIM structure (design I). Plasmonic force analysis for a PS particle (shown by black circle in part (**a**)) with the radius of 160 nm in the PV field: (**b**,**c**) Calculated plasmonic forces *F*_x_, *F*_y_, *F*_z_ and the corresponding potential wells at various *x*-offsets and *y*-offsets of the particle center, for *θ* = 45°, (**d**,**e**) Calculated plasmonic forces *F*_x_, *F*_y_, *F*_z_ and the corresponding potential wells at various *x*-offsets and *y*-offsets of the particle center, for *θ* = − 45°.

To elaborate the operation behavior of our first MIM configuration (design I) for plasmonic tweezing, the plasmonic forces exerted on the polystyrene (PS) particles with radius of 160 nm have been calculated, according to (1). Here, we have assumed *λ*_0_ = 1180 nm and laser intensity of *I*_0_ = 16.5 mW/µm^2^. First, the plasmonic forces for the presented MIM configuration with circular array are evaluated when *θ* = 45°. As it was discussed earlier in Fig. 2e–h, we have a counter-clockwise energy flow in the PV, and expect a counter-clockwise rotation of the target particle due to the exerted scattering force. Figure 3a illustrates the investigated moving direction of the target particle (black circle) in the *x–y* plane, with respect to the PV field intensity. Figure 3b,c confirm the expected rotation behavior of the PS particle for *θ* = 45°, when the particle has been swept along *x*-axis at *y* = 0 (green dashed line in part (a)), and along *y*-axis at *x* = 0 (purple dashed line in part (a)), respectively. It should be noted that the gap spacing between the particle bottom surface and the top gold layer surface is assumed equal to 20 nm in all simulations. *F*_x_ and the corresponding *U*_x_ in Fig. 3b show that the particle is trapped around the maximum field intensity in the nearly circular ring-shaped vortex, and it is rotated by the scattering force (*F*_y_) in counter-clockwise direction. Considering that the minimum potential energy is achieved about − 10 *k*_B_*T* in all parts of Fig. 3, the presented PS particle size is the minimum trappable size in our designed PV. It is well established that potential depth of about 10 *k*_B_*T* is defined as the least potential depth to overcome thermal oscillation of the trapped particles^62^. Moreover, the vertical gradient force (*F*_z_) leads to attraction of particles toward the surface of plasmonic structure, and 3D trapping of particles. The positions of maximum intensities in the ring-like PV are labeled by dashed vertical lines, and the gray zone highlights the inner effective zone of the PV in all parts of this figure. On the other hand, for *θ* = − 45° in Fig. 3d,e, energy flow of the vortex is clockwise, corresponding to the clockwise rotation of the target particle, due to the scattering force (*F*_x_), while the particle is trapped in the PV, due to the gradient force (*F*_y_) and the relating potential well (*U*_y_). Thus, we have proved successful trapping and rotating of PS particles, while by switching the linear polarization direction of the incident light from *θ* =  + 45° to − 45°, the rotation direction can be inverted from counter-clockwise to clockwise.

**Figure 4:**
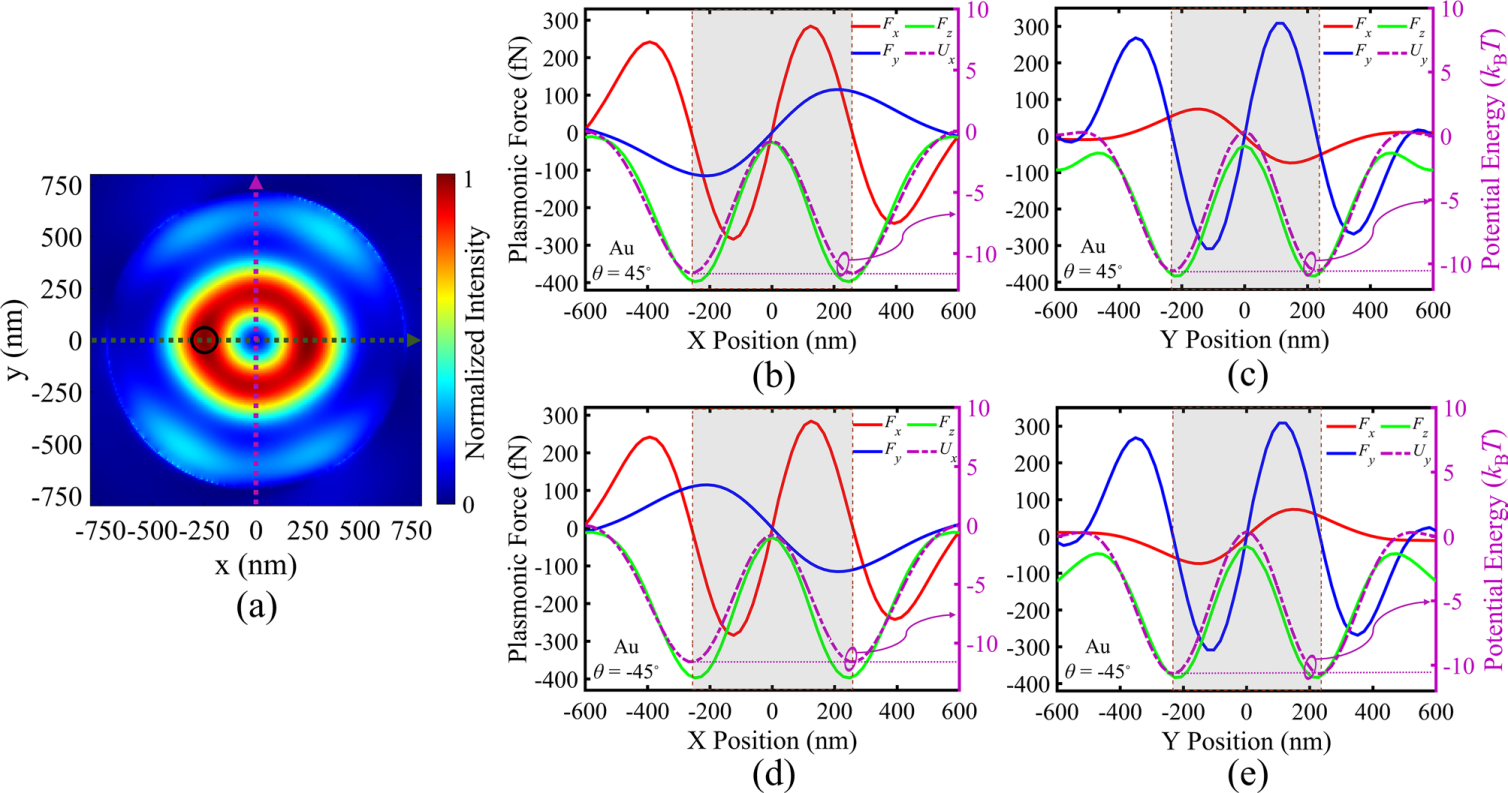
(**a**) Directions of a gold particle movements with respect to the normalized field intensity distribution of generated PV in MIM structure (design I). Plasmonic force analysis for a gold sphere (shown by black circle) with the radius of 60 nm in the PV field: (**b**,**c**) Calculated plasmonic forces *F*_x_, *F*_y_, *F*_z_ and the corresponding potential wells at various *x*-offsets and *y*-offsets of the particle center, for *θ* = 45°, (**d**,**e**) Calculated plasmonic forces *F*_x_, *F*_y_, *F*_z_ and the corresponding potential wells at various *x*-offsets and *y*-offsets of the particle center, for *θ* = − 45°.

Regarding to the strong absorption of metal particles and the exerted large scattering force, their manipulation in OVs has been challenging, while it can be achieved in PVs^51^. Thus, the trapping and rotation functionality of our designed PV are also explored for gold particles, at the same illumination conditions. For gold particles, localized plasmons of the particle can be coupled to the PV field, leading to a significant enhancement in the gradient and scattering forces. Regarding this, it is expected that smaller gold particles can be trapped and rotated at the same illumination conditions, as compared with PS particles. Accordingly, Fig. 4 indicates the same studies as Fig. 3 for a gold particle with radius of 60 nm (Fig. 4a). Figure 4b,c exhibit successful trapping and counter-clockwise rotation of the gold particle for *θ* = 45°, while Fig. 4d,e prove trapping and clockwise rotation for *θ* = − 45°. Considering the − 10 *k*_B_*T* criteria, Fig. 4b–e prove that the presented gold particle radius of 60 nm is the least trappable gold particle size in this plasmonic tweezers for *I*_0_ = 16.5 mW/µm^2^.

### Elliptical hole array design (II) with dual mode trapping/rotating operation

**Figure 5:**
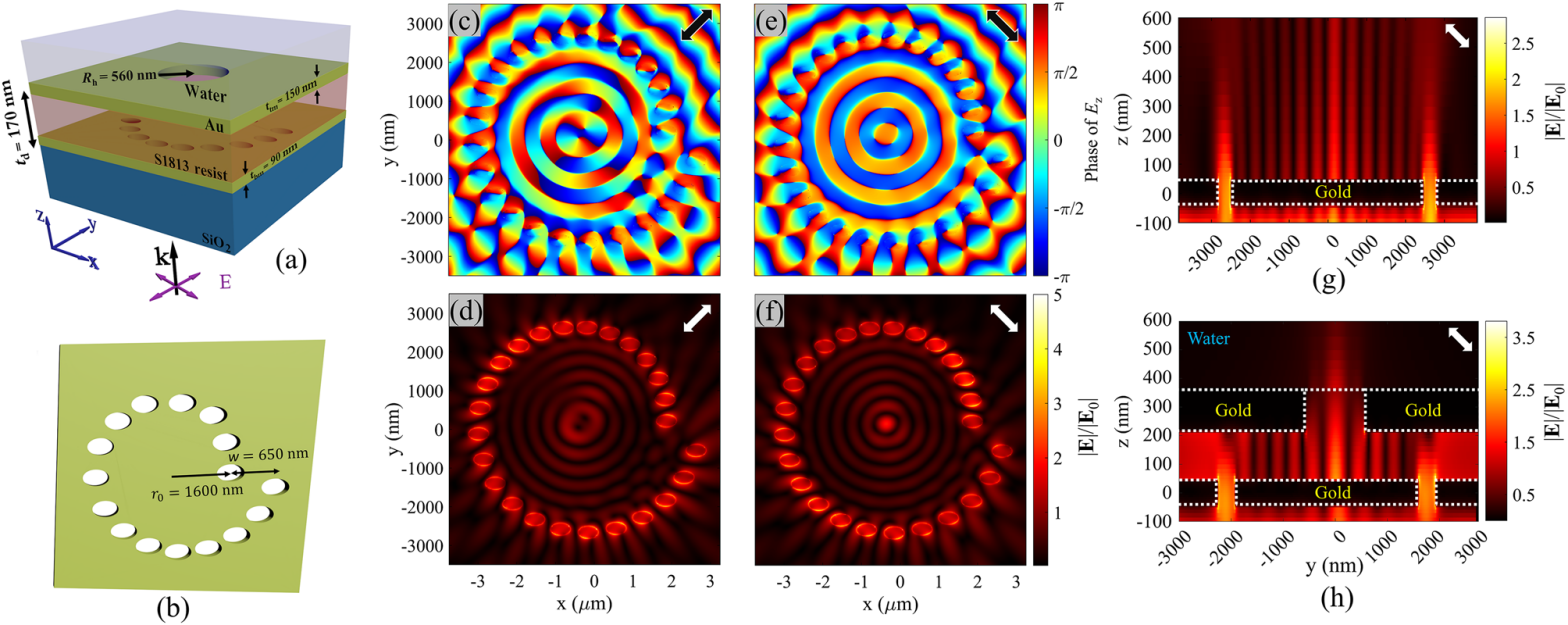
(**a**) Three-dimensional scheme of MIM structure (design II). (**b**) The schematic diagram of the array of elliptical holes with geometrical charge *m* =  + 1 embedded in the bottom gold layer in the design. In the absence of MIM configuration, and for *λ*_0_ = 1064 nm: (**c**,**d**) Phase distribution of *E*_z_ and the normalized electric field distribution, for *θ* = 45°, and (**e**,**f**) Phase distribution of *E*_z_ and the normalized electric field distribution, for *θ* = − 45°. Cross (*y*–*z* plane) views of the normalized field distribution for linear polarization with *θ* = − 45°, (**g**) in the absence, and (**h**) presence of MIM structure. Black and white arrows in parts (**c**–**h**) show *θ* orientation. Dashed white lines show boundaries of the gold layers in parts (**g**,**h**).

Figure 5a indicates the total MIM configuration based on the Archimedes spiral array as the bottom gold layer, wherein geometrical parameters are defined. The radius of the top circular hole (*R*_h_) and the top gold layer thickness (*t*_tm_) are assumed to be 560 nm and 150 nm, respectively, to achieve a high-quality PV on the top surface of MIM configuration. Figure 5b illustrates the top view scheme of the applied right-handed Archimedes spiral with *m* =  + 1, which serves as the bottom gold layer in MIM configuration. As shown in this figure, the gap between the starting point of the array to the ending point is defined as *w* = *mλ*_SPP_ in Archimedes spirals, wherein *m* is the geometrical charge of the array. First, we investigate the field intensity and phase distribution of this array under linearly polarized illumination with *θ* = 45° and *λ*_0_ = 1064 nm. The generated array PV was studied for different incident wavelengths, and the most uniform field gradient of PV has been obtained at *λ*_0_ = 1064 nm. In this case, the topological charge generated by each elliptical hole (*l* =  + 1) is added to the geometrical charge of the array (*m* =  + 1 in our design), leading to the creation of a total PV with topological charge of *q* = 2 (according to Eq. (5)). Figure 5c depicts the corresponding phase distribution of *E*_z_, while Fig. 5d indicates the relating normalized electric field amplitude of the Archimedes spiral. The simulated phase distribution for *θ* = 45° (part (c)) reveals two phase discontinuities within a 2π azimuthal rotation, which is in agreement with the expected topological charge of *q* = 2 in the designed Archimedes spiral. Again, the phase distribution and normalized electric field amplitude for *θ* = − 45° are displayed in parts (e, f) respectively. In this case, the topological charge of each elliptical hole PV is *l* = − 1 and neutralizes the geometrical charge of the array with *m* =  + 1, leading to a total topological charge of *q* = 0 (Eq. (5)). Regarding this, a uniform phase of subwavelength focusing spot is expected, which is consistent with the corresponding calculated phase distribution (Fig. 5e) and the normalized electric field amplitude (Fig. 5f). Similar to Fig. 2c, an MIM configuration based on this Archimedes spiral is designed to achieve a suitable plasmonic tweezing operation, as shown in Fig. 5a. Figure 5g shows the normalized electric field of the Archimedes spiral in the cross view (*y*–*z* plane) for *θ* = − 45°, which shows the maximum intensity on the elliptical holes, from which plasmonic waves are propagating and forming standing waves with a relative maxima in the array center. Figure 5h shows the normalized electric field of the designed MIM configuration in the cross view (*y*–*z* plane) for *θ* = − 45° and *λ*_0_ = 1190 nm, confirming successful elimination of the excessive fringes and peaks on the top gold layer surface, which is crucial for particle trapping and manipulation by the designed PV exclusively. Here, *λ*_0_ = 1190 nm has shown the best quality of the generated PV for the MIM configuration of design II. Videos of generated PVs for each design are available in supplementary files.

**Figure 6:**
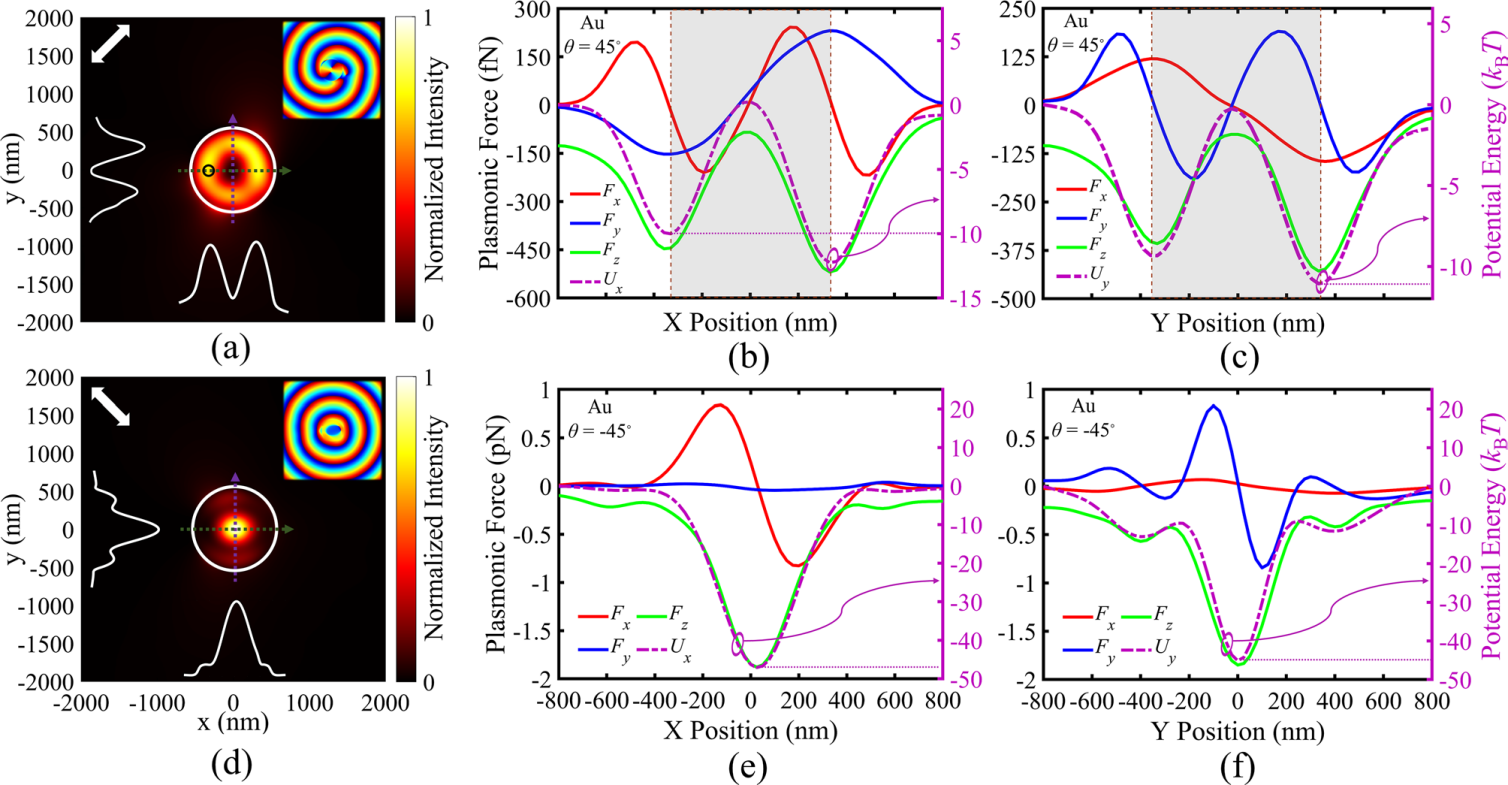
Normalized electric field intensity distribution of generated PV in MIM structure for *θ* = (**a**) 45° and (**d**) − 45°. Insets show phase distribution of *E*_z_ field in the area. Plasmonic force analysis for a gold sphere with the radius of 80 nm in the PV field: (**b**,**c**) Calculated plasmonic forces *F*_x_, *F*_y_, *F*_z_ and the corresponding potential well at various *x*-offsets and *y*-offsets along the dotted lines, respectively, for *θ* = 45°. The ring locations are labeled by dotted lines. Highlighted areas denote inside the vortex ring. (**e**,**f**) Calculated plasmonic forces *F*_x_, *F*_y_, *F*_z_ and the corresponding potential well at various *x*-offsets and *y*-offsets along the dotted lines, respectively, for *θ* = − 45°. The orientation of *θ* is shown by white arrows. Circular white lines show boundaries of the circular hole etched in the top layer.

Once again, in order to evaluate the capability of our second design (II) in rotating and trapping the target particles at the same illumination intensity (*I*_0_ = 16.5 mW/µm^2^) and *λ*_0_ = 1190 nm, we have calculated plasmonic forces exerted to a gold particle, for instance. According to Fig. 5c for *θ* = 45°, a PV with *q* =  + 2 for the final MIM configuration (design II) is expected, which is consistent with the simulated PV results in Fig. 6a. The field intensity distribution in this figure shows a single central ring-shaped hot spot, while the inset displays the relating phase distribution of *E*_z_ with *q* =  + 2. Now, the calculated plasmonic forces on a gold particle with radius of 80 nm (shown by black circle) are plotted in Fig. 6b,c, while it is swept once along *x*-axis at *y* = − 18 nm (green dashed line), and once along *y*-axis at *x* = − 16 nm (purple dashed line), respectively. It is observed that the total PV in design II shows a slight off-center position, due to the slight asymmetry geometry in the Archimedes spiral with *m* = 1. The achieved potential profiles along *x*, and *y*-axes reveal dual potential wells with depths of about 10 *k*_B_*T* on the ring-shaped PV, proving a stable and successful trapping. Moreover, the calculated *F*_y_ in part (b), and *F*_x_ in part (c) behave as the scattering forces, leading to counter-clockwise particle rotation for the case of *θ* = 45°. Then, changing the polarization angle to *θ* = − 45°, Fig. 6d reveals a single central circular hotspot, and the inset shows the relating phase distribution of *E*_z_ with *q* = 0. Figure 6e,f display the calculated plasmonic force components, corresponding to sweeping the particle along *x*-axis at *y* = 21 nm (green dashed line), and along *y*-axis at *x* = 9 nm (purple dashed line), respectively. It is worth mentioning that the calculated scattering forces, *F*_y_ in part (e) and *F*_x_ in part (f) are negligible for *θ* = − 45°, which are consistent with *q* = 0, and proving an exclusive trapping behavior in this mode. The achieved potential well shows a single central potential well with depth of about − 45 *k*_B_*T,* exceeding from the minimum requirement for stable trapping. It is notable that for this trapping mode, the illumination intensity can be reduced down to 3.4 mW/µm^2^, and decrease the potential depth to 10 *k*_B_*T*. However, considering both operation modes in this design (II), the minimum potential depth corresponds to the case of *θ* = 45° (Fig. 6b,c), which defines the minimum required illumination intensity for stable trapping operation.

### Elliptical hole array design (III) with PV direction independent of incident polarization angle

**Figure 7:**
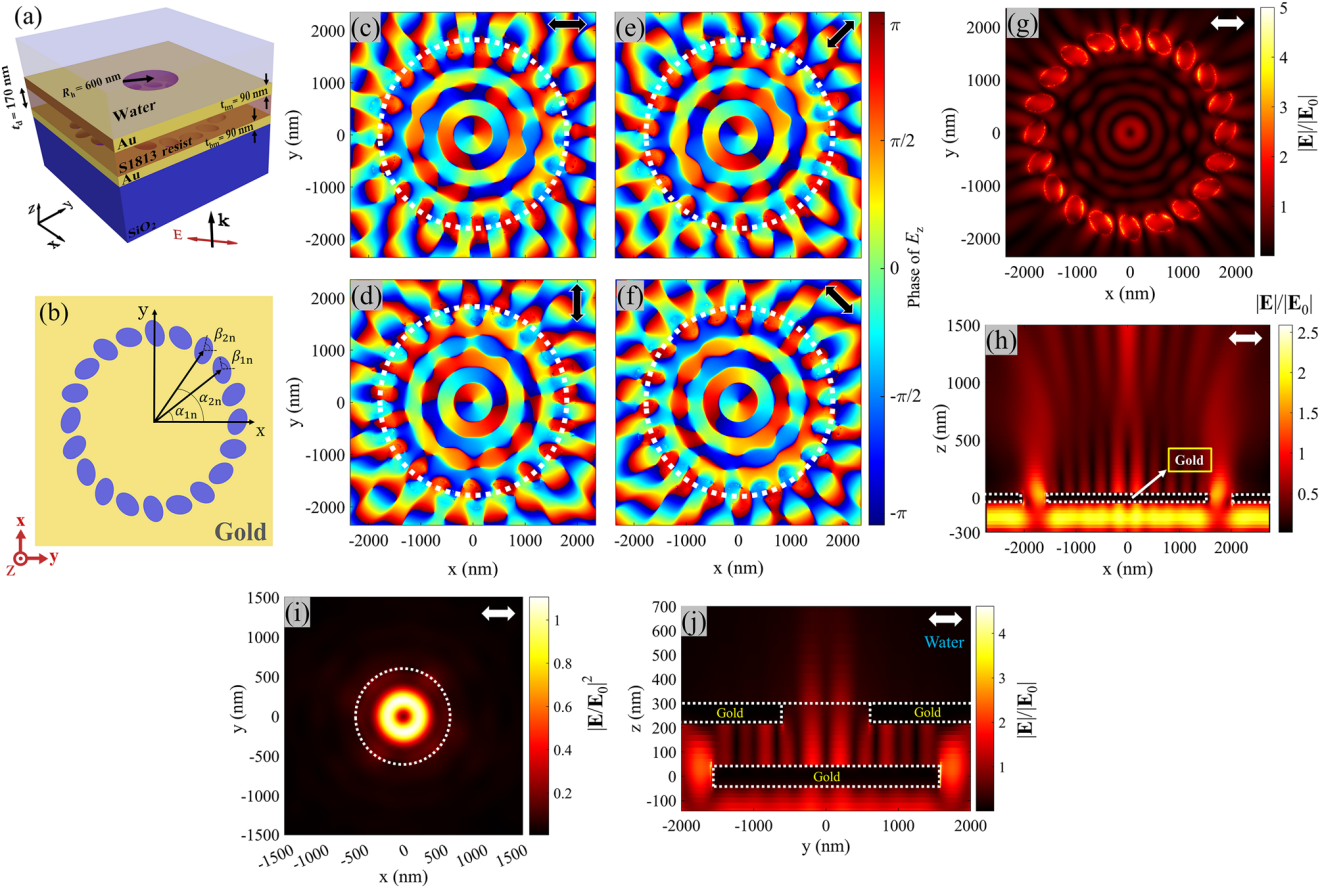
(**a**) Three-dimensional schematic of MIM structure and (**b**) The schematic diagram of the polarization independent array of elliptical holes embedded in the bottom gold layer. (**c**–**f**) Phase distribution of *E*_z_ for *θ* = 0°, 90°, 45° and − 45°, respectively. Dashed lines indicate the location of holes position (**g**,**h**) Normalized electric field distribution in *x*–*y* plane and *x*–*z* plane, respectively for *θ* = 0°. (**i**) Normalized electric field intensity distribution at *x*–*y* plane and (**j**) Normalized electric field amplitude distribution in *y*–*z* plane, in MIM configuration for *θ* = 0°. White dashed lines illustrate the gold layer boundaries. White and black arrows indicate orientation of linear polarization.

Here, we design an elliptical hole array, capable of producing PV when illuminated by linearly polarized incident light with arbitrary orientation. The proposed array geometry is similar to a previous report based on rectangular hole array in silver layer, in which an efficient PV has been produced, independent of the polarization angle of the linearly-polarized illumination^40^. As shown in Fig. 7a, an MIM configuration is proposed based on a circular array with radius of 1800 nm as the bottom layer. Figure 7b exhibits the top view of this circular array with 20 elliptical holes (*N* = 20), consisting of two sets with the same number of holes being equal to 10. It is evident in this figure that two neighboring elliptical holes in our array belong to different sets, in this design. As illustrated in Fig. 7b, *α*_*in*_ indicates the position angle of the *n*th hole in each set, while *β*_*in*_ stands for the rotation angle of the corresponding hole with respect to the *x*-axis. *n* changes from 1 to 10, while *i* = 1, 2 representing the first and second hole sets. Wang et al. proved that the geometrical parameters (*α*_*in*_*, β*_*in*_) of this array are correlated as follows^40^

6 $${\alpha }_{1n}=2n\pi /N$$

7 $${\beta }_{1n}={\alpha }_{1n}+{\alpha }_{1}$$

8 $${\alpha }_{2n}=\left(2n+1\right)\pi /N$$

9 $${\beta }_{2n}={\alpha }_{2n}+{\alpha }_{2}$$

wherein *α*_1_ and *α*_2_ are assumed to be 60° and 30° in our design, respectively. It can be proved from Eqs. (6–9) that two neighboring elliptical holes lead to one equivalent PV with topological charge of + 1, independent of *θ*. So, 10 of these neighboring elliptical hole pairs can be arranged in a circular array to achieve a high-quality PV independent of *θ* with *q* =  + 1^40^. In this array with different orientations of the elliptical holes, plasmonic fields can be excited at the holes under different polarization angles of incident light, that propagate towards the array center and interfere to form a high-quality central PV, independent of *θ*. First, the generated array PV was studied for different incident wavelengths, achieving the most uniform field gradient of PV at *λ*_0_ = 850 nm for this array configuration (see Fig. S2). Utilizing the same elliptical holes geometry in different array structures have imposed different array geometry constraints, leading to a small tolerance in the designed array radii in our three designs (I, II, III). However, small tolerance in array radii can lead to a significant phase difference in the propagating plasmonic waves at the array center, which can affect the quality of the equivalent PV significantly. This fact has led to a significant change in the optimum operation wavelength of the latter array in design III, as compared to our two former designs. To explore the polarization angle independency, the array is simulated for illuminations at the wavelength of *λ*_0_ = 850 nm, and different polarization angles. In Fig. 7c–f, the phase distribution of this array is plotted for polarization angles (shown by black arrows) of *θ* = 0°, 90°, 45° and − 45°, respectively. As observed, high quality PVs with *q* =  + 1 are generated independent of polarization angle in this elliptical hole array, but the initial phase is different for different *θ* values. Figure 7g illustrates the top view (*x*–*y* plane) normalized amplitude of the electric field distribution relating to *θ* = 0°, at 10 nm above the metal surface. It is observable in parts (c–g) that both phase and field amplitude distributions show higher circular symmetry than our former array designs (Figs. 2, 5). Moreover, the achieved high circular symmetry ring-shaped field amplitude at the array center (Fig. 7g) benefits from higher field amplitude than the former designs, which is suitable for efficient particle manipulation and is attributed to better excitement of all elliptical holes in this array, independent of *θ*. However, it was observed in Figs. 2b and 5d,f that some elliptical holes were excited stronger than the others, depending on the polarization angles. Moreover, Fig. 7h exhibits the cross-section view (*x*–*z* plane) of the normalized field amplitude distribution at *y* = 0 nm, and for *θ* = 0°, as an example case. Now, to generate a single central PV and screen the undesired side peaks of field intensity, again an MIM configuration is proposed as shown schematically in Fig. 7a. It can be observed that we assumed the radius of the top circular hole, the gold layers thicknesses, and S1813 thickness equal to 600 nm 90 nm and 170 nm, respectively. Figure 7i,j show the top and cross section views of the relating electric field intensity and amplitude distribution, when *θ* = 0° and *λ*_0_ = 1047 nm proving a single circular PV, suitable for particle manipulation. Here, *λ*_0_ = 1047 nm has shown the best quality of the generated PV for the MIM configuration of design III. It can be observed that the proposed MIM design (III) benefits from a strong gradient field in addition to efficient and uniform rotational mode for any linear polarization orientation. It is worth noting that this array can also be arranged to generate any other values of topological charges independent of *θ.* For example, if rotation angle of holes correlated as ${\beta }_{1n}={\alpha }_{1n}-{\alpha }_{1}$ and ${\beta }_{2n}={\alpha }_{2n}-{\alpha }_{2}$, a PV with topological charge of − 1 can be generated independent of *θ* (see Fig. S3). Moreover, if the holes are arranged in Archimedes spiral array (Eq. (3)) simultaneously, different values of topological charges are achievable, independent of *θ* (see Fig. S4).

**Figure 8:**
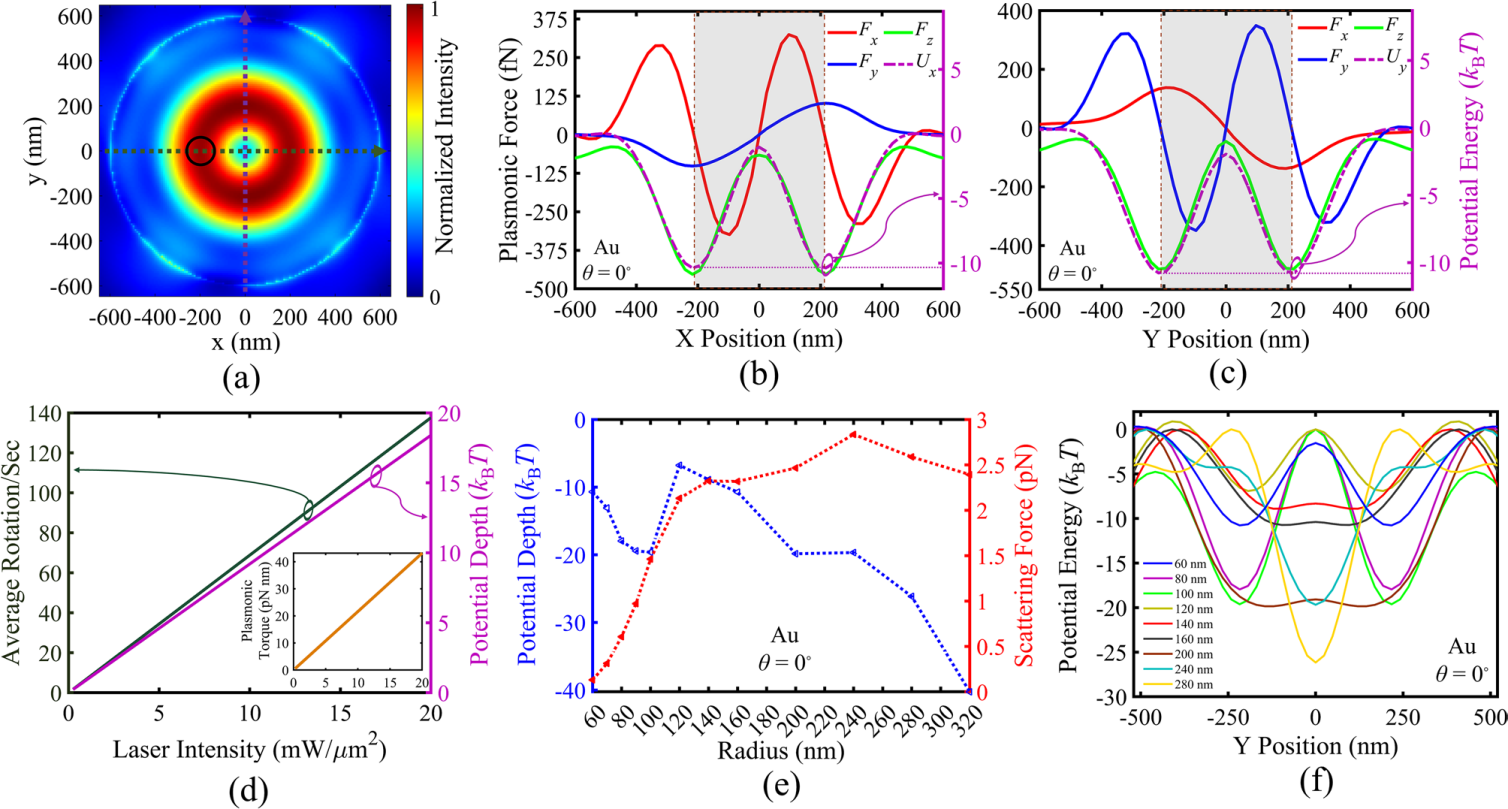
(**a**) Normalized electric field intensity distribution of generated PV in MIM structure, and direction of particles movement. Plasmonic force analysis for a gold sphere (shown by black circle) with the radius of 60 nm in the PV field: (**b**,**c**) Calculated plasmonic forces *F*_x_, *F*_y_, *F*_z_ and the corresponding potential well at various *x*-offsets and *y*-offsets along the dashed lines, respectively, for *θ* = 0°. The ring locations are labeled by brown dashed lines. Highlighted areas denote inside the vortex ring. (**d**) Rotational speed, Potential depth and plasmonic torque of a 60 nm gold particle as a function of illumination intensity. (**e**,**f**) Calculated scattering forces and the corresponding potential well along *y*-offsets for different sizes of gold particles for *I*_0_ = 11.5 mW/µm^2^.

Here, to elaborate on the plasmonic tweezing behavior of this design (III), regarding the trapping and rotating ability of the achieved PV, we calculate the plasmonic force components exerted on the gold particle with radius of 60 nm, as an example target particle. The illumination wavelength, power intensity and polarization angle are assumed to be *λ*_0_ = 1047 nm, *I*_0_ = 11.5 mW/µm^2^, and *θ* = 0°. Here, a lower illumination power density has been required to achieve the − 10 *k*_B_*T* criteria and trapping the gold particles with the same size as design I (60 nm), owing to the achieved PV with higher field amplitude and gradient force in design III. As shown in Fig. 8a, the particle has been swept in the vicinity of the centered PV, once along *x*-axis at *y* = 0 nm (green dashed line), and along *y*-axis at *x* = 0 nm (purple dashed line). The calculated plasmonic force components and the corresponding potential profiles are depicted in Fig. 8b,c. It is obviously observed in these parts that the particle is being trapped by the strong gradient forces, while the azimuthal plasmonic scattering forces give rise to a counter-clockwise rotation of particle. It is notable that due to the small chamber dimensions (~ 1 mm) in lab-on-chip microfluidics and the usual slow convection flow velocity (~ 10 µm/s), the Reynolds number in these systems is very small (~ 0.01) revealing a Laminar flow condition. It is well known that rotational speed of a rotating particle equals *v*/2π*r*_v_, wherein *r*_v_ is the rotational radius or our PV radius, and *v* is the particle velocity. Here, rotational speed of the trapped particle with radius of *R* in fluid media with viscosity of *η*, can be estimated by equating the drag force *F*_D_ = 6π*ηRv* to the calculated azimuthal scattering force. In Fig. 8d, both the calculated rotational speed and potential depth are plotted for the investigated gold particle with radius of 60 nm in the achieved PV versus illumination power density. The observed linear behavior for both curves in this figure reveals that the trapping and rotation behavior of our designed PV can be controlled by illumination power linearly. Moreover, it can be observed that illumination power density of about 11 mW/µm^2^ is the minimum required power for stable trapping of this particle, leading to a rotational speed of about 70 rounds/s. It should be noted that some experimental reports have been achieved rotational speeds about 8 times smaller than this simplified theoretical estimation^63^. The reported retarded rotational speed can be attributed to underestimation of drag coefficient, and possible non-uniformity in the localized field intensities, and the relating gradient and scattering forces, and the Brownian movements of the trapped particles^64^. Moreover, the exerted threshold plasmonic torque can be estimated by *τ* = *r*_v_ × *F*_D_, where *r*_v_ is the radius of the rotation track or the PV radius. The inset in Fig. 8d indicates the estimated plasmonic torque exerted to the similar gold particle versus power density, showing a linear behavior.

To explore the effect of target particle size on the trapping and rotating behavior of our final PV design (III), gold particles with different radii are inspected, while *I*_0_ = 11.5 mW/µm^2^. Figure 8e shows that for *R* < 100 nm, the larger particles have led to the higher gradient and scattering plasmonic forces. Otherwise, for *R* > 120 nm the profile of potential depth declines sharply. Figure S5 shows the normalized electric field around the gold particles of different radii in the vicinity of the PV, which confirms the fact that plasmonic behavior of the gold particles in the vicinity of PV changes significantly around the so-called “nano-scale” dimension of about 100 nm. Moreover, Fig. 8f illustrates that by increasing *R* more than about the PV radius (*r*_v_ = 210 nm in design III) the potential profile converts from dual well profile to a single well profile with a centered valley, while scattering forces decrease smoothly simultaneously. These observations are attributed to the fact the particles larger than about the PV diameter interact with the PV as a whole equivalent hot spot at larger distances from the gold layer surface. Moreover, both the opposing-directed scattering forces affect on a single large particle, leading to some extent of force cancelation. This investigation is also performed for different sizes of PS particles in Fig. S6. However, it should be noted that due to the larger (negative and lossy) refractive index of gold, the induced surface plasmons on the gold particles strongly interact with the PV, which has led to stronger scattering forces, as compared with the PS counterparts.

Here, plasmonic tweezers based on elliptical holes array have been designed and simulated, which can be controlled by the incident polarization angle and allow more uniform and smoother rotating functionality, as compared with previously reported plasmonic counterparts such as rectangular and cross-shaped holes^39,40^. Despite other works on tweezers^43–50^, we have demonstrated different modes of particle manipulation such as trapping, clockwise and counter-clockwise rotation. Moreover, both gold and PS particles with sizes below 160 nm are successfully trapped and rotated in our generated PVs, while in other works the presented size of rotating target particles is around 1 μm^33,51^. This work has also been optimized by an MIM configuration to eliminate any interfering excessive fields, which has not been reported in previous plasmonic tweezers designs, making this design feasible for application in integrated lab-on-chip systems.

## Conclusions

We have provided the numerical demonstration of three independent ultracompact plasmonic devices being able to produce surface plasmon waves possessing pure orbital angular momenta of preassigned orders. In particular, the device converts impinging light with linear polarization to a near-field plasmonic vortex, which is attractive for application in plasmonic tweezers. The structure consists of a planar MIM waveguide implemented on a glass substrate. The bottom gold layer is arranged with an array of elliptical holes to generate a PV, and the top gold layer screens all perturbations emanating from localized plasmonic fields of the elliptical cavities, while an embedded central circular hole in this layer allows transmission of the generated PV exclusively. Initial vortex modes corresponding to each elliptical hole can be generated, when the direction of the incident polarization differs from the main ellipse axes. We have designed three different structures with dissimilar arrays of elliptical holes through the bottom gold layer. The first design can produce counter-clockwise and clockwise rotating plasmonic vortex under polarization angle of 45° and − 45°, respectively. The second one can produce the PV with topological charges of 0 and + 2, depending on the orientation of linearly polarized light. The third one can generate a robust counter-clockwise rotating plasmonic vortex under arbitrary direction of incident linear polarization. Next, it is proved that target particles can be trapped and manipulated in such vortices in accordance with the designed characteristics of the PVs. Both gold and polystyrene particles are investigated in the designed plasmonic tweezers. We have used gold particles in exposure to PVs to confirm the advantage of PVs versus conventional OVs for manipulating metallic particles. Our simulations have shown that the particle will be trapped in the vortex ring by the gradient force, and then is pushed to rotate by the scattering force. To the best of our knowledge, rotation of particles of sub-wavelength dimensions about *λ*_0_/20 for gold particles, and *λ*_0_/8 for polystyrene particles, had never been reported. We believed that our study opens new perspective in manipulating subwavelength scale particles, while proposing a high degree of freedom in manipulation. It is expected that our device can also be used for manipulation of dielectric particles with larger sizes or higher refractive indices. Our design can be used in compact lab-on-a-chip systems, wherein controllable rotation or trapping of different target particles are needed. For instance, trapping and rotating particles to induce vortices in the fluid for the purpose of local mixing, or utilizing the rotational force to apply torque to the biological instances such as proteins and DNA to investigate their properties.

## Supplementary Information

Supplementary Figures.

## Data Availability

The datasets used and/or analyzed during the current study available from the corresponding author on reasonable request.
