# Supplementary material for: Plasmonic vortices for tunable manipulation of target particles, using arrays of elliptical holes in a gold layer
Source: Sci Rep. 2023 Jan 2;13:54. doi: 10.1038/s41598-022-27109-7 (PMC9807555; doi:10.1038/s41598-022-27109-7)
Supplement: Supplementary file 1 — Supplementary Figures. [file 41598_2022_27109_MOESM1_ESM.docx]

**Supporting Information for:**

Plasmonic Vortices for Tunable Manipulation of Target Particles, Using Arrays of Elliptical Holes in a Gold Layer


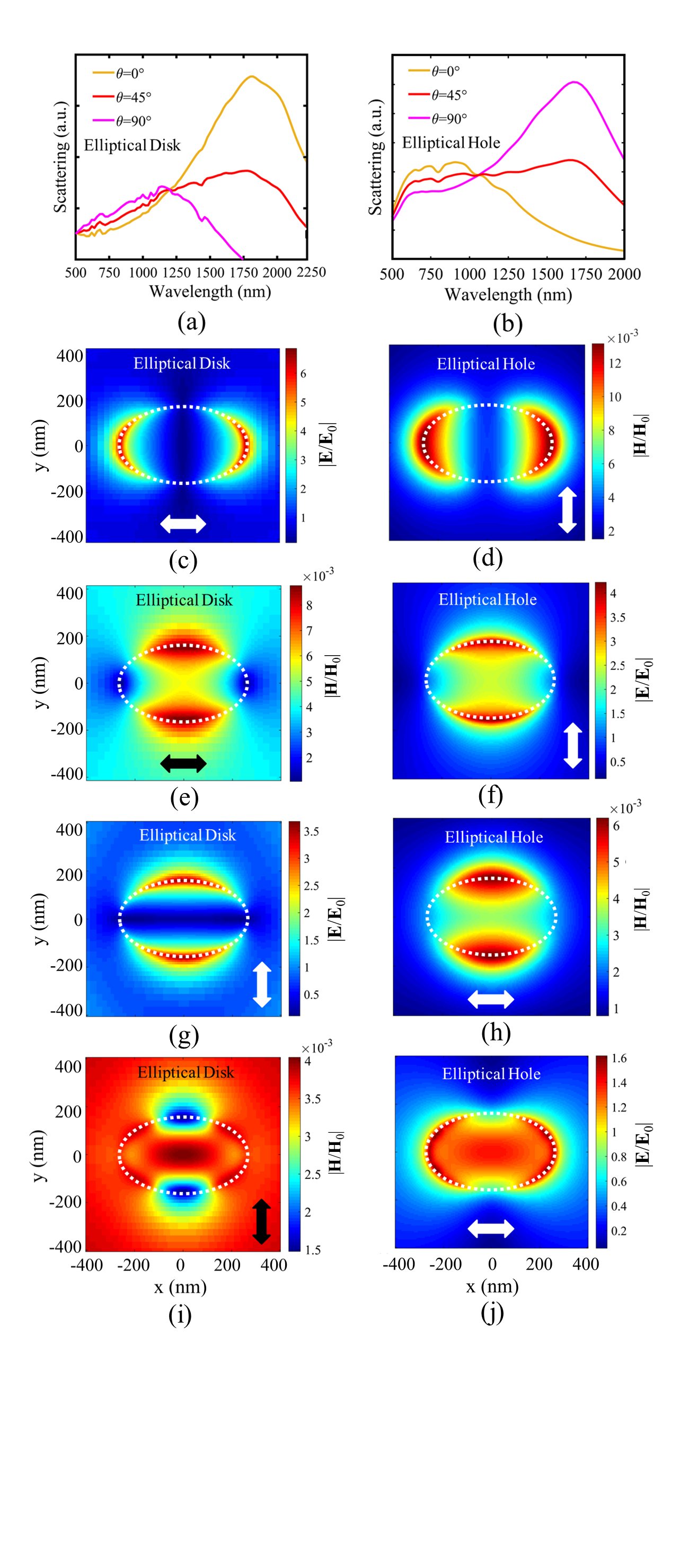


**Figure S1.** (**a**) The scattering spectrum of a single elliptical golden disk measured in different θ values of 0^⸰^, 90^⸰^ and 45^⸰^, excited from the substrate. Major axis and minor axis size of the disk are 560 nm and 320 nm, respectively. (**b**) The scattering spectrum of a single elliptical hole etched in the gold layer measured in different θ values of 0^⸰^, 90^⸰^ and 45^⸰^, excited from the substrate. Major axis and minor axis size of the hole are 560 nm and 320 nm, respectively. (**c**) Normalized electric field distribution in x-y plane around the single elliptical disk for θ = 0^⸰^ and λ_0_ = 1800 nm. (**d**) Magnetic field distribution in x-y plane around the single elliptical hole for θ = 90^⸰^ and λ_0_ = 1700 nm. (**e**) Magnetic field distribution in x-y plane around the single elliptical disk for θ = 0^⸰^ and λ_0_ = 1800 nm. (**f**) Normalized electric field distribution in x-y plane around the single elliptical hole for θ = 90^⸰^ and λ_0_ = 1700 nm. (**g**) Normalized electric field distribution in x-y plane around the single elliptical disk for θ = 90^⸰^ and λ_0_ = 1150 nm. (**h**) Magnetic field distribution in x-y plane around the single elliptical hole for θ = 0^⸰^ and λ_0_ = 1047 nm. (**i**) Magnetic field distribution in x-y plane around the single elliptical disk for θ = 90^⸰^ and λ_0_ = 1150 nm. (**j**) Normalized electric field distribution in x-y plane around the single elliptical hole for θ = 0^⸰^ and λ_0_ = 1047 nm. White dashed lines illustrate the boundary of elliptical hole or disk , and White and black arrows indicate the orientation of linear polarization.


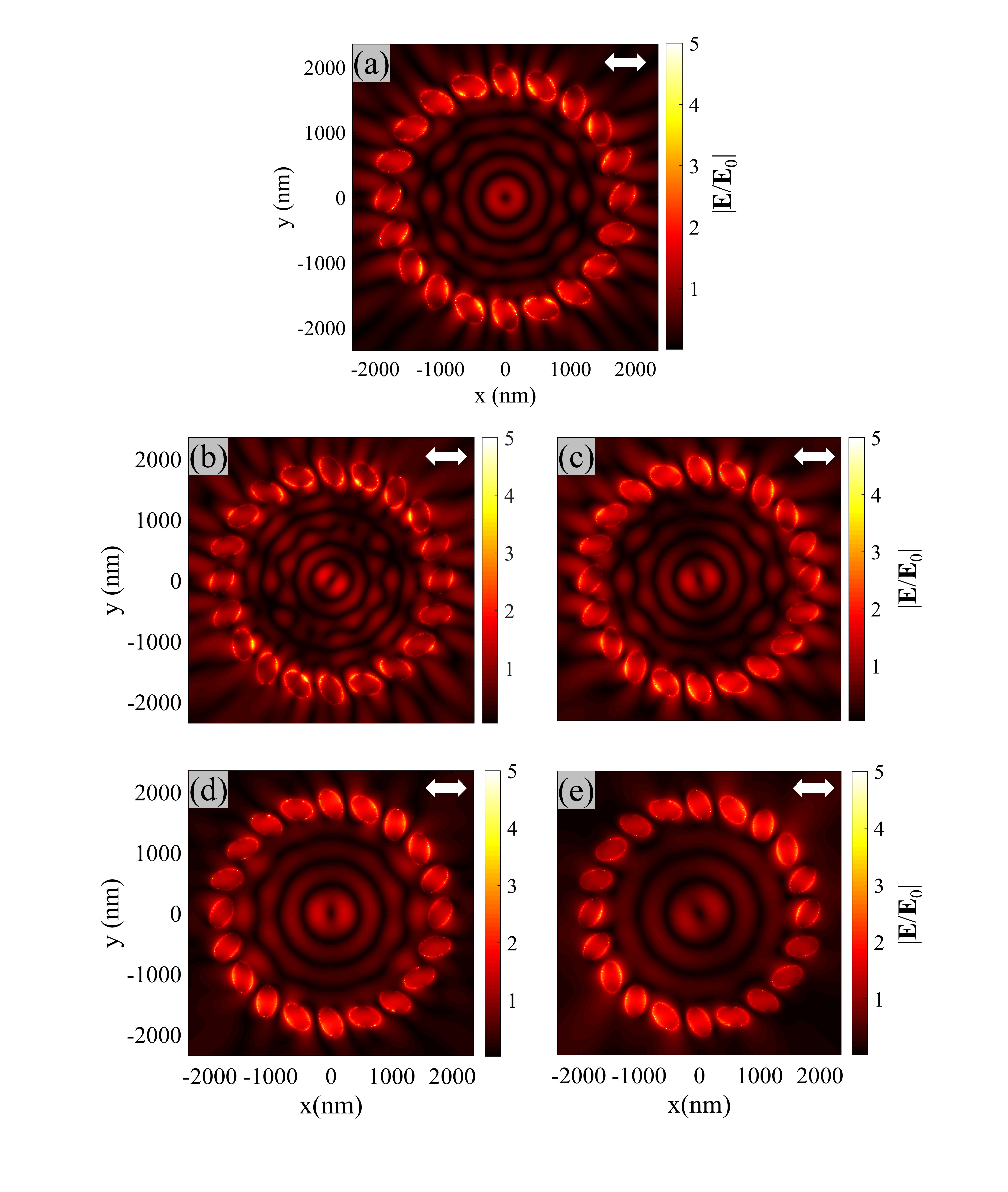


**Figure S2.** (**a-e**) Normalized electric field distribution in x-y plane for array design (III) for λ_0_ = 850 nm, λ_0_ = 760 nm, 930 nm, 1064 nm and 1240 nm, respectively. White arrows indicate orientation of linear polarization.


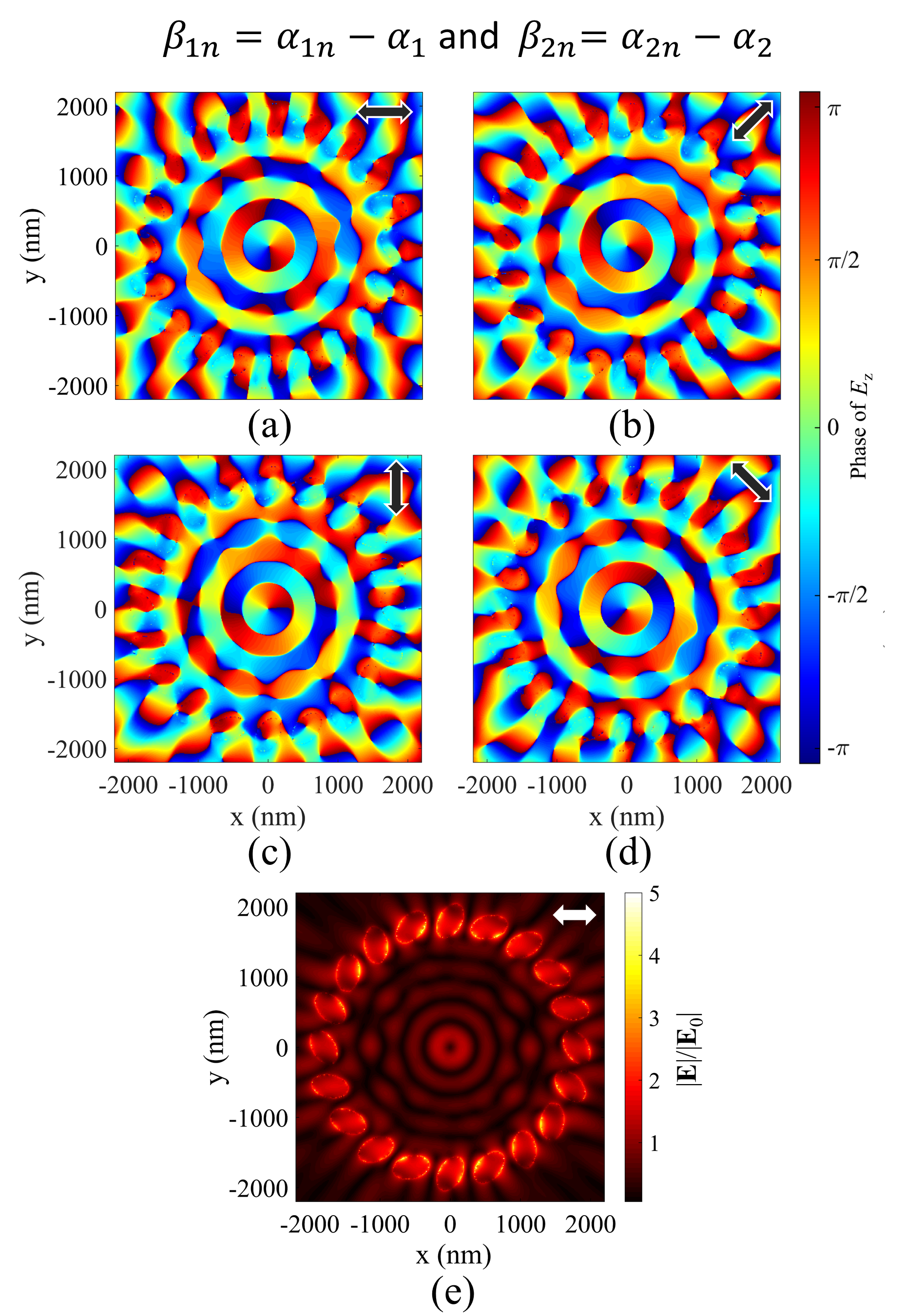


**Figure S3.** (**a-d**) Phase distribution of E_z_ in the array of design (III), when the rotation angles of the holes satisfy $\beta_{1n}=\alpha_{1n}-\alpha_{1}$ and $\beta_{2n}=\alpha_{2n}-\alpha_{2}$, for θ = 0^⸰^, 45^⸰^, 90^⸰^ and -45^⸰^, respectively. (**e**) Normalized electric field amplitude distribution in x-y plane for θ = 0^⸰^ in this design. The arrows indicate orientation of linear polarization.


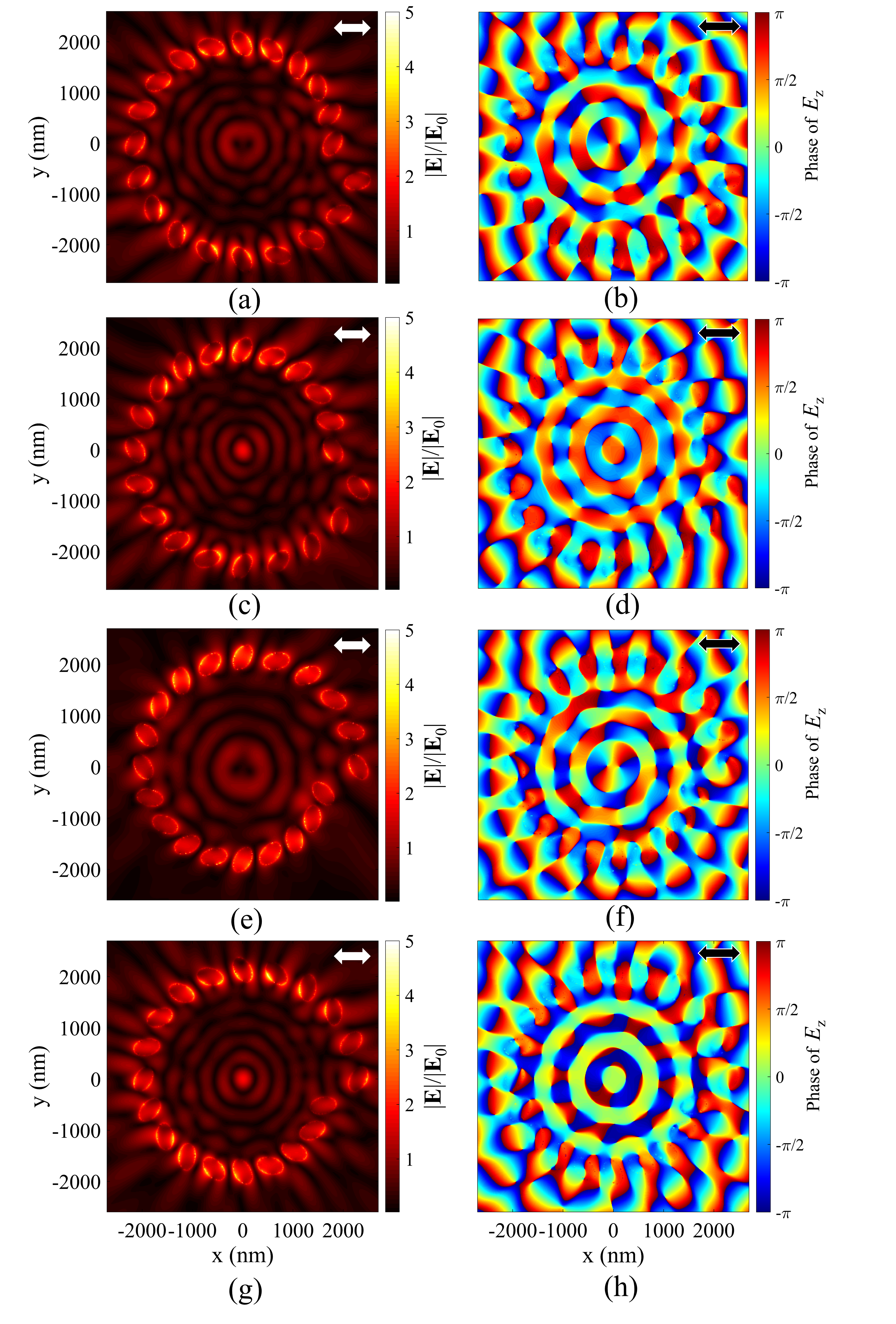


**Figure S4.** (**a, b**) normalized electric field distribution and Phase distribution of E_z_ field, respectively, when the holes arrangement satisfy $\beta_{1n}=\alpha_{1n}+\alpha_{1}$, $\beta_{2n}=\alpha_{2n}+\alpha_{2}$ and $r\left( \emptyset\right)=r_{0}+(m\emptyset\lambda_{SPP})/2\pi$ in which m = 1. In this case, a PV with q = 2 is produced independent of θ. (**c, d**) normalized electric field distribution and Phase distribution of E_z_ field, respectively, when the holes arrangement satisfy $\beta_{1n}=\alpha_{1n}-\alpha_{1}$, $\beta_{2n}=\alpha_{2n}-\alpha_{2}$ and $r\left( \emptyset\right)=r_{0}+(m\emptyset\lambda_{SPP})/2\pi$ in which m = 1. In this case, a PV with q = 0 is produced independent of θ. (**e. f**) normalized electric field distribution and Phase distribution of E_z_ field, respectively, when the holes arrangement satisfy $\beta_{1n}=\alpha_{1n}-\alpha_{1}$, $\beta_{2n}=\alpha_{2n}-\alpha_{2}$ and $r\left( \emptyset\right)=r_{0}-(m\emptyset\lambda_{SPP})/2\pi$ in which m = -1. In this case, a PV with q = -2 is produced independent of θ. (**g, h**) normalized electric field distribution and Phase distribution of E_z_ field, respectively, when the holes arrangement satisfy $\beta_{1n}=\alpha_{1n}+\alpha_{1}$, $\beta_{2n}=\alpha_{2n}+\alpha_{2}$ and $r\left( \emptyset\right)=r_{0}-(m\emptyset\lambda_{SPP})/2\pi$ in which m = -1. In this case, a PV with q = 0 is produced independent of θ. White arrows indicate orientation of linear polarization, and λ_0_ = 915 nm.


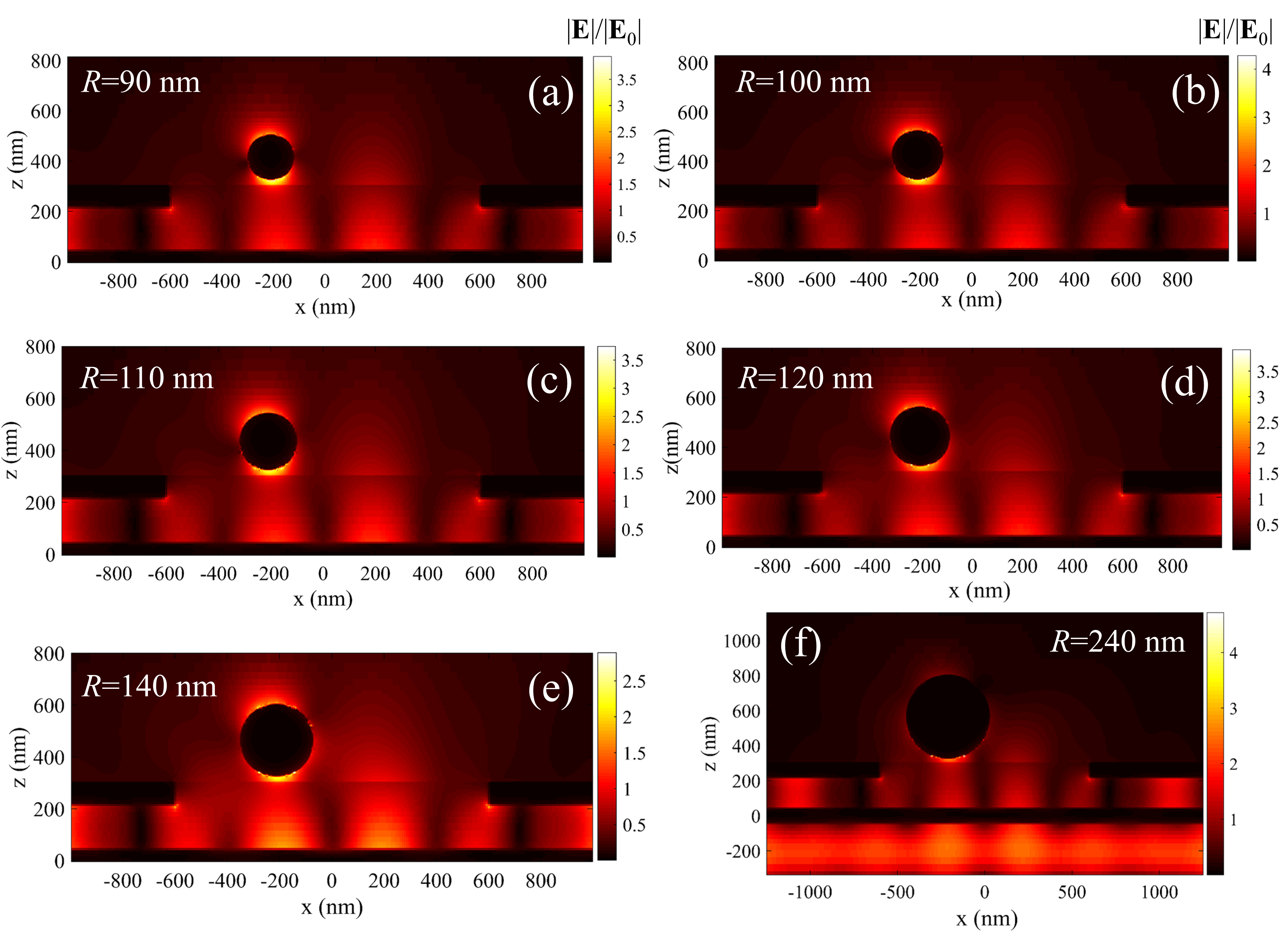


**Figure S5.** (**a-f**) normalized electric field around gold particles with radius of 90 nm, 100 nm, 110 nm, 120 nm, 140 nm and 240 nm, respectively, in the vicinity of the PV.

Here, we explore the effect of PS particle size on the trapping and rotating behavior of our final PV design (III) for *I*_0_ = 15 mW/µm^2^. Figure S6a shows in the investigated size range, the larger particles have led to the higher gradient and scattering plasmonic forces, which are consistent with the well-established approximations in Rayleigh regime (*R*<*λ*), including $F$*_Grad_* $\propto R^{3}$ and $F$*_Scatt_* $\propto R^{6}$ [1]. It can be observed in part (b) that by increasing *R* more than about the PV radius (*r*_v_ = 210 nm in design III) the potential profile converts from dual well profile to a single well profile with a centered valley. Also, scattering forces begin to decrease smoothly at the similar particle size. These observations are attributed to the fact the particles larger than about the PV diameter interact with the whole PV and experience an equivalent single hot spot at larger distances from the gold layer surface. Moreover, both the scattering forces with opposite direction affect on a single large particle, leading to some extent of rotational force cancelation.


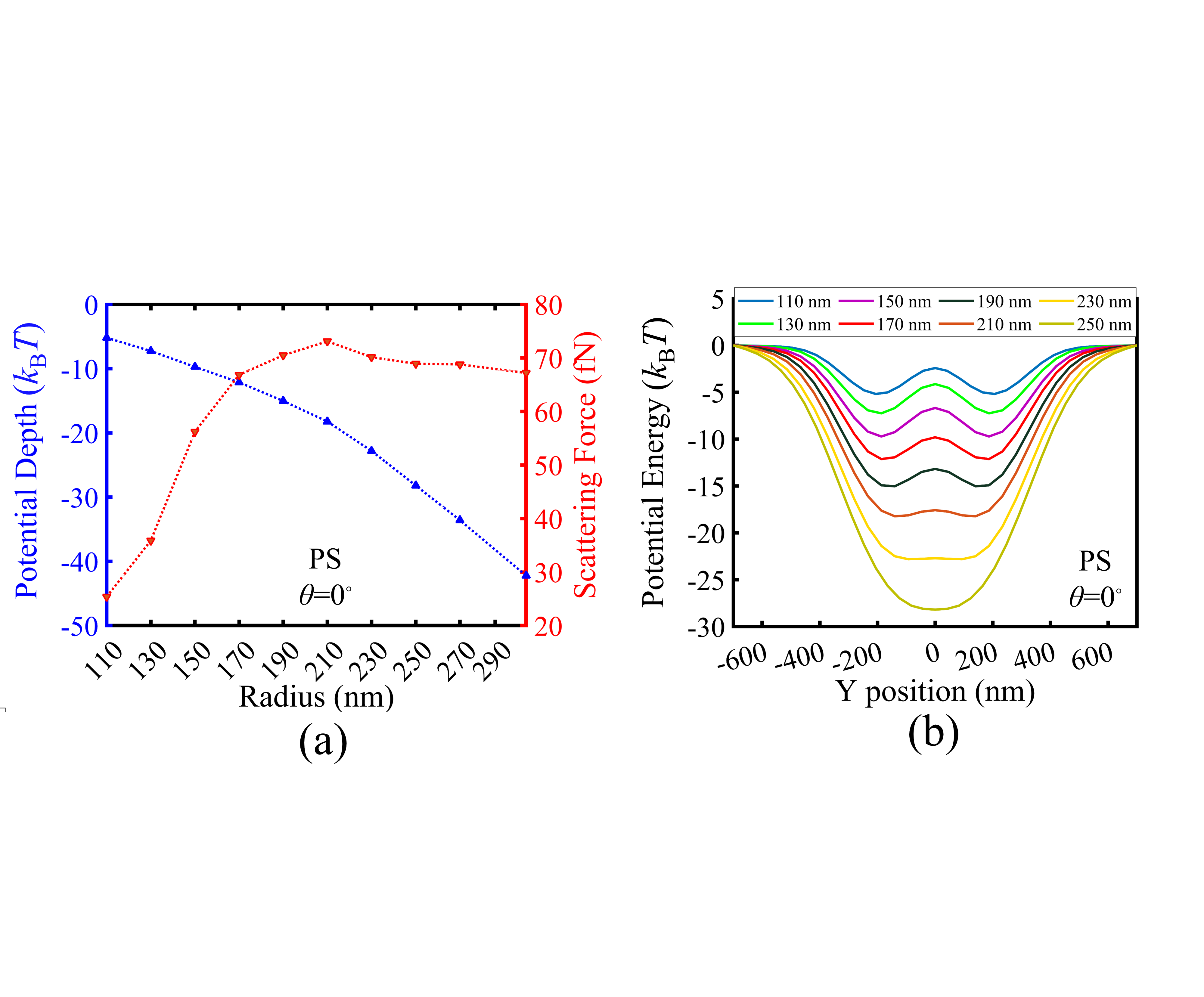


**Figure S6.** (**a, b**) Calculated scattering forces and the corresponding potential well along y-offsets for different sizes of PS particles for I_0_ = 15 mW/µm^2^.

**References**

1. Svoboda, K. & Block, S.M. Optical trapping of metallic Rayleigh particles. *Opt. Lett.* **19**, 930-932 (1994).
